# Supplementary material for: Patient experience of a virtual reality calm room in a psychiatric inpatient care setting in Sweden: a qualitative study with inpatients
Source: BMJ Open. 2023 Dec 14;13(12):e076285. doi: 10.1136/bmjopen-2023-076285 (PMC10729110; doi:10.1136/bmjopen-2023-076285)
Supplement: Author's manuscript [file bmjopen-2023-076285.draft_revisions.pdf]

BMJ Open is committed to open peer review. As part of this commitment we make the peer review history of every article we publish publicly available.

When an article is published we post the peer reviewers' comments and the authors' responses online. We also post the versions of the paper that were used during peer review. These are the versions that the peer review comments apply to.

The versions of the paper that follow are the versions that were submitted during the peer review process. They are not the versions of record or the final published versions. They should not be cited or distributed as the published version of this manuscript.

BMJ Open is an open access journal and the full, final, typeset and author-corrected version of record of the manuscript is available on our site with no access controls, subscription charges or pay-per-view fees (http://bmjopen.bmj.com).

If you have any questions on BMJ Open's open peer review process please email info.bmjopen@bmj.com

Patient experience of a virtual reality calm room in a psychiatric inpatient care setting: a qualitative study

Journal:	*BMJ Open*
Manuscript ID	bmjopen-2023-076285
Article Type:	Original research
Date Submitted by the Author:	02-Jun-2023
Complete List of Authors:	Ilioudi, Maria; University of Gothenburg Sahlgrenska Academy, Institute of Health and Care Sciences<br>Wallström, Sara; Institute of Health and Care Sciences, Sahlgrenska Academy, University of Gothenburg; University of Gothenburg Centre for Person-Centred Care, Sahlgrenska Academy, University of Gothenburg<br>Steingrimsson, Steinn; Sahlgrenska universitetssjukhuset, Psychiatric Department; University of Gothenburg Institute of Neuroscience and Physiology, University of Gothenburg<br>Lindner, Philip; Karolinska Institutet, Centre for Psychiatry Research, Department of Clinical Neuroscience<br>Thunström, Almira Osmanovic; University of Gothenburg Institute of Neuroscience and Physiology<br>Ali, Lilas; Health and Care Sciences, Sahlgrenska academy
Keywords:	Virtual Reality, PSYCHIATRY, MENTAL HEALTH

SCHOLARONE™
Manuscripts

I, the Submitting Author has the right to grant and does grant on behalf of all authors of the Work (as defined in the below author licence), an exclusive licence and/or a non-exclusive licence for contributions from authors who are: i) UK Crown employees; ii) where BMJ has agreed a CC-BY licence shall apply, and/or iii) in accordance with the terms applicable for US Federal Government officers or employees acting as part of their official duties; on a worldwide, perpetual, irrevocable, royalty-free basis to BMJ Publishing Group Ltd ("BMJ") its licensees and where the relevant Journal is co-owned by BMJ to the co-owners of the Journal, to publish the Work in this journal and any other BMJ products and to exploit all rights, as set out in our licence.

The Submitting Author accepts and understands that any supply made under these terms is made by BMJ to the Submitting Author unless you are acting as an employee on behalf of your employer or a postgraduate student of an affiliated institution which is paying any applicable article publishing charge ("APC") for Open Access articles. Where the Submitting Author wishes to make the Work available on an Open Access basis (and intends to pay the relevant APC), the terms of reuse of such Open Access shall be governed by a Creative Commons licence – details of these licences and which Creative Commons licence will apply to this Work are set out in our licence referred to above.

Other than as permitted in any relevant BMJ Author's Self Archiving Policies, I confirm this Work has not been accepted for publication elsewhere, is not being considered for publication elsewhere and does not duplicate material already published. I confirm all authors consent to publication of this Work and authorise the granting of this licence.

For peer review only

Patient experience of a virtual reality calm room in a psychiatric inpatient care setting: a qualitative study

Authors: Maria Ilioudi[1,2], Sara Wallström[1,3,4], Steinn Steingrimsson[1,5], Philip Lindner[6], Almira Osmanovic Thunström[1,5], Lilas Ali[1,2,3]

Correspondence to: Assoc. Prof. Steinn Steingrimsson, MD, PhD, Department of Psychiatry, Sahlgrenska University Hospital, Region Västra Götaland, 41650 Gothenburg, Sweden; Tel: 46 313421000: Email: steinn.steingrimsson@neuro.gu.se

Manuscript type: Original research

Abstract length: 287

Text length: 4246

No. of references: 58

No. of graphics: 3 (2 tables/1 figure)

For peer review only

ABSTRACT

Objective Calm rooms have been developed and implemented in psychiatric inpatient care settings to offer patients a dedicated space for relaxation in a convenient and safe environment. Recent technology developments have enabled virtual reality (VR) equivalents of calm rooms that can be feasibly deployed in psychiatric care settings. While research has shown VR environments to be efficacious in inducing relaxation, little is known how these virtual calm rooms are perceived by patients. The aim of this study was to elucidate patient experiences of using a VR calm room in a psychiatric inpatient setting.

Design Qualitative interview study. Semi-structured interviews were analysed using qualitive inductive content analysis, which focuses on the interpretation of texts for making replicable and valid inferences.

Setting Swedish hospital psychiatric inpatient care setting with a wireless, three degrees-of-freedom VR head-mounted display running a calm room application simulating nature environments.

Participants 20 adult patients (12 women) with bipolar disorder (n=18) or unipolar depression (n=2).

Results Participants experienced the use of the VR calm room as having a positive impact on them, inducing awareness, calmness and well-being. They were thankful to be offered a non-pharmacological alternative for anxiety relief. Participants also expressed that they had some concerns, at first, about how they would react emotionally before using the VR device; however, after use, they highlighted that their overall experience was positive. They also expressed that they could see potential for further development of VR technology in psychiatric care.

For peer review only

Conclusions VR technology has the potential to solve pressing logistic issues in offering calm rooms in psychiatric inpatient care. VR calm rooms appear to be appreciated by psychiatric inpatients, who value their accessibility, convenience and variety of modalities offered. Participants perceived an increase in their well-being after use.

Keywords: virtual reality, psychiatry, relaxation, psychiatric inpatient care, qualitative design, interviews

For peer review only

For peer review only - http://bmjopen.bmj.com/site/about/guidelines.xhtml

Strengths and limitations of this study

- This study was conducted in an inpatient psychiatric setting with fully representative patients with severe psychiatric conditions who were introduced to a VR calm room as a new method for relaxation.
- The interview data in this study provided rich descriptions of patient experiences of using the VR calm room.
- The sample was broad, interviewing women and men with a wide age range.
- There were some difficulties in engaging patients in longer interviews, which was not unexpected given the acute phase that patients were in at that time.
- The short duration of engagement by the informants during interviews resulted in more inclusions (n=20) to have enough data to draw necessary conclusions.

For peer review only

INTRODUCTION

There is ample evidence showing that exposure to nature settings such as trees, parks and beaches induces a sense of well-being, at least in the short-term.[1] Relaxation interventions have been explored as alternatives to nature experiences in various healthcare settings worldwide.[2] Previous studies have indicated that participation in different forms of relaxation such as meditation, yoga, muscle relaxation, abdominal breathing and guided imagery induce a state of general well-being, including feelings of calmness and being in a pleasant mood.[3] Moreover, relaxation exercises have also been shown to decrease muscle tension, blood pressure, heart rate and respiration rate.[3–5]

Mental health conditions are by definition associated with unpleasant feelings, for which relaxation may offer temporary relief, and this has been thoroughly explored in the literature.[6–8] Relaxation techniques used in stress management have been shown to be effective, posing low risks and being easily taught.[9 10] Managing mental health conditions requires a variety of strategies and previous research has largely focused on traditional relaxation techniques.[5]

Calm rooms in both physical[11] and virtual reality (VR)[12] format have been introduced into psychiatric inpatient settings over the last decade. The purpose is to offer patients their own space for relaxation in order to induce well-being through calming exercises and distraction from distress.[13–15] Calm rooms can also be used to provide self-help through a convenient and safe environment and, at the same time, empower the therapeutic relationship between the patient and healthcare workers at inpatient units.[11 16] Previous research indicates that calm rooms, both in physical and VR format, can be used for clinical purposes as a beneficial method for patients in reducing stress and inducing well-being.[11 14 15 17 18]

For peer review only - http://bmjopen.bmj.com/site/about/guidelines.xhtml

VR technology is increasingly being used in the treatment of mental disorders and numerous interventions have been developed and tested.[19–23] The term "virtual reality" has been used to describe a computer-generated, three-dimensional and gaze-interactive environment.[24] The VR environment enables relaxation through visualisation, engagement and immersion into pleasant virtual environments, and by removing users from stressful situations.[5]

There are some clinical studies involving VR calm rooms in psychiatric settings which show the efficacy of VR environments for mental health conditions.[18 25–27] Studies have also shown the efficacy[28–30] of VR in treating mental conditions, including social anxiety disorder,[31 32] post-traumatic stress disorder,[33] generalised anxiety disorder[18] and phobias.[21 34] Furthermore, VR technology has been used to provide immersive experiences for relaxation.[2 35–38] A previous study has suggested that VR nature relaxation immediately reduced negative affective states and psychological stress in patients with psychiatric disorders.[27]

VR environments provide a digital ecosystem and present a paradigm shift in the availability of VR therapies in mental health.[19] In addition, VR technology solves logistic issues since it can be used anywhere[26 29] and is affordable in comparison to refurbishing a room at a psychiatric inpatient care setting.[21] The technology has also been shown to be convenient and safe in psychiatric inpatient care settings since it is made of harmless materials and is usually used with supervision until the patient is trusted on his or her own.[40] VR relaxation settings provide a sense of presence, pleasure, activation and engagement, and a personalised experience.[41]

There is, however, a lack of knowledge of how users in psychiatric inpatient care perceive the use of VR-based relaxation technologies. The aim of this study,

For peer review only - http://bmjopen.bmj.com/site/about/guidelines.xhtml

therefore, was to elucidate patient experience of using a VR calm room in psychiatric inpatient care.

MATERIAL AND METHODS

Study design

This study has a qualitative and descriptive design. Semi-structured interviews were conducted and analysed using a qualitive content analysis based on inductive theory. This study was part of a larger research project aimed at evaluating the effect of VR and physical calm rooms in psychiatric inpatient care.[12]

Setting and participants

A total of 20 patients (12 women and 8 men) participated in the study. There was no overlap in samples between the current study and a previously reported trial.[12] At inclusion, the participants were admitted for psychiatric inpatient care at a hospital in the western region of Sweden (Sahlgrenska University Hospital, Gothenburg). Participant characteristics are summarised in table 1. Inclusion criteria were age ≥18 years, admitted to the inpatient psychiatric care setting specialised in bipolar disorder and having previously used the VR calm room. Due to a lack of empty beds in other settings, patients with other psychiatric diagnoses were also included in this study.

For peer review only - http://bmjopen.bmj.com/site/about/guidelines.xhtml

Table 1 Patient demographic and clinical characteristics (n=20)

Variable	No. of patients
Sex	
Men	8
Women	12
Age, years	
20–29	4
30–49	5
50–59	5
60–69	6
Diagnosis	
Bipolar disorder	18
Unipolar depression	2

Procedure

Patients who used the VR calm room received instructions on the VR headset's functions and how to navigate in the virtual environment. To engage with the device, patients used a wireless, three degrees-of-freedom VR head-mounted display (an Oculus Go) running the Calm Room application developed by Mimerse. Example screenshots of the environment are shown in figure 1. The application offers a variety of soothing nature environments that can be tailored through, for example, day and night scenes and dynamic weather. In addition, it includes breathing exercises, mindfulness programs and relaxing music. The user can interact with the environment by looking at different objects inside the program and using the handheld controller to adjust preferences and build individualised scenery.

Data collection

The individual interviews all followed a semi-structured interview-guide developed by the authors. The interviews were conducted between October 2021 and May 2022 by the first author (MI). Interviews took place at the psychiatric inpatient care setting during patients' care time. The mean duration of the interviews was 15.15 (range,

For peer review only

10–24) minutes. The interviews were recorded and transcribed verbatim. The participants were asked, for example, how they experienced the calm room, was there anything that worried them when using the VR calm room, how do they think an alternative to the VR calm room can be developed and what kind of difference do they think using the VR calm room has made for their situation.

Data analysis

Data were analysed using qualitative inductive content analysis, which focuses on the interpretation of texts for making replicable and valid inferences.[42] The inductive approach entails that text is analysed based on the participants' narratives through grouping meaning units containing aspects related to each other in content and context.[43] Baxter[44] defines 'meaning unit' as the constellation of words or statements that relate to the same central meaning and argues that themes are threads of meaning that recur in domain after domain. A theme is a thread of an underlying meaning through meaning units or codes on an interpretive level. A theme can be seen as an expression of the latent content of the interview text.[44] The success of content analysis depends on reaching reliability and validity through integrity of the coding process.[45]

First, the first and last authors (MI and LA) familiarised themselves with the material by reading the transcriptions several times in order to grasp the central meaning of the texts. Through careful reading, relevant sentences and phrases were identified. These were then used in the constellation of meaning units and to begin the coding process. All authors discussed the labelled codes, which were formed into themes and further divided into sub-themes.

For peer review only - http://bmjopen.bmj.com/site/about/guidelines.xhtml

Ethical considerations

Ethical approval was granted by the Swedish Ethical Review Authority (Dnr: 318-18). All participants received verbal and written information about the study and that they could end the interview at any time without specifying a reason. All data were treated confidentially. All ethical decisions were guided by the Declaration of Helsinki[46] and its subsequent amendments The participants admitted to the psychiatric inpatient care setting may have been under compulsory psychiatry care. The authors were aware that the participants' mental health condition might be affected and were prepared to interrupt the research process or offer suitable support services upon participant request or if this was deemed necessary.

RESULTS

The first overall impression of the analysis revealed that using the VR calm room had a positive impact on the participants during their period of care at the inpatient psychiatric unit. The experience raised feelings of awareness, calmness and well-being. Participants also had suggestions on further development of the VR technology in mental healthcare in the future. An overview of the themes and sub-themes is presented in table 2.

Table 2 Themes and sub-themes from interviews following VR calm room use

Feelings before and during use	Negative point of view	Potential for future development
Being offered an alternative to medication	Practical issues and side effects	Willing to continue use
Feelings of relief and relaxation	Unpleasant feelings	Recommending VR
Sense of independency		

For peer review only - http://bmjopen.bmj.com/site/about/guidelines.xhtml

Feelings before and during use

Participants expressed that they had a positive attitude about using the calm room before the intervention and thought it would be an enjoyable part in the overall time of care at the psychiatric inpatient unit. Several participants mentioned that they preferred non-pharmacological alternatives such as VR. Furthermore, they experienced a sense of independence and gave positive feedback about the opportunity to learn new relaxation methods.

Being offered an alternative to medication

During inpatient psychiatric care, the participants sometimes encountered boundaries in being able to achieve a peaceful state of mind on their own due to the care environment being hectic or perceived as stressful. They perceived there to be few non-pharmacological alternatives available or any support offered to them to enhance their well-being and reduce anxiety. They therefore expressed gratitude over being offered the use the VR device as an alternative to drugs.

The participants also mentioned that use of the virtual calm room was very anxiety-relieving and therefore had a positive impact on their well-being during the time they were admitted to the inpatient psychiatry unit. Being admitted to psychiatric inpatient care was typically associated with high levels of anxiety and feelings of seclusion. The participants highlighted that inpatient psychiatric care needs alternative methods that they can be offered for anxiety reduction and self-support. The participants also stated that they were upset about the limited options as well as the traditional relaxation methods previously available and therefore really appreciated the time that they spent in the VR calm room. The VR calm room enriched their day at the psychiatric inpatient care setting by giving them a new

For peer review only - http://bmjopen.bmj.com/site/about/guidelines.xhtml

dimension to rely on when needed. The participants described the VR rooms as a strategy with which they could find respite and develop emotional self-care in a stressful environment at the psychiatric inpatient care setting. Participants also highlighted that the VR represented something innovative in psychiatric care which gives hope for new treatment options.

> "…one thing is that we have a lot of time here, it's slow, you have nothing to do, you maybe feel bad, if it's anxiety. That you get a tool and you sit for a certain time. I think that can help, because sometimes you might just go and go and go and do nothing, then it doesn't get better, there are no…you have to have multiple tools. This…virtual room, well, I think it's a good thing." P1

For peer review only

Feelings of relief and relaxation

Participants had varying experiences of how VR could potentially influence their mental condition in a better way. The experiences of use were described as being helpful for their condition and that it gave them a sense of a holistic approach to their feelings. They expressed feelings of joy and that it was something beautiful to experience. Participants also stated that using the VR calm room fulfilled their day because they felt that they had done something creative and worthwhile for their condition. They described the environment they experienced in the VR device as dynamic and that they found some kind of a new dimension to strategically enhance their well-being. Participants also experienced the VR environment to induce a state of calm and creating an awareness of their emotions at the time of use. They found it comfortable to go into another reality and leave the physical one behind for a while.

Participants in this study usually experienced a stressful reality on a daily basis which contributed to their feelings of anxiety.

"...it felt very nice to enter another world and then that I felt calmer afterwards." P1

Sense of independency

The VR device was viewed as an opportunity for participants to learn new relaxation methods, such as relaxing the body, practicing breathing and maintaining positive thinking. Participants expressed that it was easy to learn and practice guided meditation using the VR application. Furthermore, they mentioned that VR is easier to handle compared to usual relaxation methods that they would use in the "real world". Participants wished to stay longer in the VR calm room because they described their use as a new way to reach a feeling of calmness. Participants also expressed that using the VR room gave them a sense of independence in the psychiatric inpatient care setting by allowing them to make their own decisions and becoming more involved in their own care.

"It's not easy to describe. Now I'll see, you first came...completely shocked by that nature and then feelings just grow, it's nice inside you that fills up the soul." P7

Participants indicated that the device was comfortable and flexible to use. They could, on their own, decide how they wanted their calm room to be and the duration of the session. This was all handled by themselves, which gave them a sense of independence in the psychiatric inpatient unit. Furthermore, participants expressed that being able to stay in the room at the psychiatric inpatient care setting solved

For peer review only - http://bmjopen.bmj.com/site/about/guidelines.xhtml

logistic issues and could therefore be used at any place in the ward based on their own individual preferences. They also appreciated that they could pause, end and restart the session at any time.

> "If you're alone, if no staff is here, the VR glasses would have been good because then you can also lie down. I thought that was good. In this relaxation thing, they don't nag. In some relaxation videos it's, tie the hand, open the hand, tie, open. Here it was just, tie the hand and then you did the rest yourself. So that was enough information." P5

Negative point of view

This theme describes the participants' negative experiences and feelings related to the use of the VR device. Some experienced unpleasant feelings at the start when first using the VR calm room. They were afraid and worried about some components of the VR nature environment such as the darkness and birds. Some of the participants also mentioned that the device was not very comfortable to wear on their head.

Practical issues and side effects

Participants experienced negative issues with the VR device being uncomfortable and heavy to carry for a longer period of time on their head. Some participants mentioned that the VR device did not fit properly and would therefore slip during the time they used it. This affected the overall experience of VR device use. They also mentioned that sometimes they would become dizzy and feel nauseous while using the device. Participants also mentioned that these issues usually came at the start of

For peer review only - http://bmjopen.bmj.com/site/about/guidelines.xhtml

the session and would become less common as they became more familiar with the VR environment. None of the participants interrupted their session due to cybersickness. Before using the VR device, they had their hesitations, some of them worried that they might feel a bit dizzy and uncomfortable. However, they managed to overcome that, after using the VR device for a while, finding it was not as frightening or strange as they thought that it would be. The participants expressed that, when they used the VR device, they felt more relaxed at the end of the session than before.

> "...but that it slipped and then it got on the glasses and then there was no sharpness so I had to take it up all the time..." P3

Unpleasant feelings

Entering the VR world gave some of the participants unpleasant feelings such as sometimes being stressed and worried for no reason that they can recall. They were also expecting their own imagination to kick in and that is why they did not want to be left alone in the VR calm room. They indicated that they were waiting for more life and interaction in the virtual world such as with animals or other creatures.

> "At first it was a bit strange and scary because when I turned my head there was like nothing but the VR environment and then I got stressed at first because I felt it was too close and I didn't recognise myself. But then it got better and then it became a bit like my environment, my own. So it turned around and got better..." P5

For peer review only - http://bmjopen.bmj.com/site/about/guidelines.xhtml

Furthermore, the analysis indicated that some of the participants experienced VR as frightening and an unsafe environment when specific images would appear such as birds flying everywhere or something else unexpected that might happen in the VR environment making them stay alert in case something unexpected would occur. Participants also described that one scenario in the VR was too dark, which usually led to them becoming anxious of the light and therefore feeling uncomfortable as it would remind them of walking into death when you see the light at the end of the session.

"....I perceived the light as...a bit like death, that you walk towards the light, that it became too much...that it became too peaceful..." P4

"Then the birds, there were so many birds, it didn't feel like a threat but it felt a bit stressful, I don't like seagulls, so it got a bit like this..." P15

Potential for future development

This theme captures participants' suggestions and recommendation on future use of a VR calm room and what kind of potentials they see for further development, for example, VR as an alternative to treatment for stress reduction.

Willing to continue use

The VR experience was described as a new experience for the participants. They stated that they would consider using the VR device again both in a healthcare setting and also by themselves for self-care at home. They wanted to have easy accessibility to the VR device every day in the psychiatric inpatient care setting and

For peer review only - http://bmjopen.bmj.com/site/about/guidelines.xhtml

wished that there would be several VR devices available for use. Furthermore, the participants expressed that they wished to use VR on a daily basis as a part of their treatment. Several participants wanted to have a VR device of their own and asked if they could buy one or get access to the calm room application. The main statement that was given by the participants in this study was that they wanted to use the device again in the future.

"I want to use it again and I was still sceptical before, I was open-minded, sceptical, but I would like to use it again if I get the chance..." P20

Recommending VR

Participants mentioned that VR could reduce anxiety and induce well-being. They recommended the use of the VR device for therapeutic purposes as they could see potential for that. Despite some frightening features, the participants also described use as a positive and not harmful experience, which is why they would recommend it to others.

"It's relaxing so it affects everyone positively, it can be positive for everyone..." P7

However, there were some participants who mentioned that they thought that the VR concept might be inappropriate for patients who have vision problems and difficulties with crisis situations, such as for some patients with psychosis. It might also be inappropriate to use when feeling too tired or trapped. Participants wished for further functions to be developed in the VR device and also wanted to be able to choose different environments, not just the natural calm room that was programmed.

For peer review only - http://bmjopen.bmj.com/site/about/guidelines.xhtml

They wanted to experience more dynamics, and visions that included more animals and other creatures than what they had experienced. The participants recommended and wished to use the VR for about 15 to 20 minutes as they felt that in that time they could experience the full effect of anxiety relief.

DISCUSSION

The aim of this study was to elucidate experiences of using a VR calm room by patients admitted to psychiatric inpatient care. Our findings strengthen the results of previous research, which showed VR calm rooms to increase well-being and lower distress.[2 36 38 47] In the current study, participants expressed feelings of relaxation, which induced their well-being, when using the VR room. Implementation of VR technology in psychiatric inpatient care settings has been shown to successfully improve patient recovery and emotional self-care[48] as a VR calm room strengthens patient choices, leading to a flexible and convenient strategy for resolving distress in an acute psychiatric setting.[12] Such methods might enhance patient engagement in their care and to take on the responsibility for recovery of their severe mental condition.

The findings support that the VR calm room reduces the experience of stress and helped patients to feel better with a sense of calm. Previous studies have shown that immersion into VR environments has positive psychological and physical effects.[27 49] Previous research has found that a VR calm room design can distract patients from stressful acute psychiatric settings.[27 50] The design of the VR room in our study, which aimed at creating a less hospital-like experience with features supporting familiarity and flexibility, may have contributed to the patients' sense of safety, self-control and enjoyment. Exposure to VR nature images can provide emotional well-

For peer review only - http://bmjopen.bmj.com/site/about/guidelines.xhtml

being for patients who do not have access to outdoor activities and nature.[51] Patients stay in psychiatric inpatient care for long periods of time and VR calm rooms have the potential to offer visual access to nature and its positive effects. VR technology may solve logistics issues in the psychiatric inpatient care as patients can use the VR device in their own room in the psychiatric inpatient setting without affecting safety.[19 52] In comparison to traditional relaxation methods, participants mentioned that the VR calm room was easy to access and use. VR relaxation is appreciated by users,[53] and has become affordable and more accessible, which has led to its increasingly widespread use in psychiatric care.[50]

Participants reported several negative experiences with the use of VR. Patients expressed unpleasant feelings and being worried for no reason. They also mentioned becoming dizzy and that some of the images in the VR calm room were scary. The unpleasant feelings might be due to the new environment and unfamiliar components. Our findings align with previous studies where users reported issues with the installation process, overheating, the visual presentation and negative effects such as anxiety.[19] It is possible that some of these negative experiences may be due to personal preferences, which might be avoidable by offering the user a variety of environments.[54] Furthermore, patients admitted to psychiatric inpatient care experience low satisfaction with their influence on the choice of treatment and medication.[55] Non-pharmacological methods such as physical calm rooms have safety issues; therefore, healthcare workers may become risk averse and initially worried about letting patients stay on their own in calm rooms.[11 56] However, activities that break up the monotony in psychiatric inpatient care settings were appreciated by the patients.[57]

For peer review only - http://bmjopen.bmj.com/site/about/guidelines.xhtml

The participants in this study highlighted some future recommendations and suggestions about VR calm rooms such as creation of several scenarios and device options with more modalities. This is in line with previous research which suggests that the VR scenario should be expanded to include various additional experiences such as live music scenes, scenes featuring people walking around and animals.[58] Patients recommended the use of the VR device as a therapeutic method for relaxation and stress reduction both in healthcare settings and for patients to use on their own at home. However, some participants mentioned that VR might be inappropriate for patients with vision problems and when being too tired. Also, some recommended avoiding the use of VR in crisis psychiatric conditions due to the high levels of anxiety and lower adaptation ability in these circumstances. Self-relaxation is an important feature of the care process in psychiatric inpatient care; therefore, participants stated that they wanted to use the VR room in the future as a relaxation tool. Broader use of VR calm rooms should be investigated to explore their potential for being part of usual treatment in acute psychiatric care.

Study strengths and limitations

The primary strength of this study is that it was conducted in inpatient psychiatric care, with fully representative patients. It is especially important to include patients who are diagnosed with severe acute psychiatric conditions as they are often excluded from studies due to their illness. This study had a sample of participants with bipolar diagnosis and unipolar depression. The interview data in this study provided rich descriptions of patient experiences of using the VR calm room. Moreover, we reached a broad population sample by interviewing women and men of different ages. The qualitative approach using content analysis[42–44] made it

For peer review only - http://bmjopen.bmj.com/site/about/guidelines.xhtml

possible to elucidate patient experiences using a VR calm room in acute psychiatric care. Trustworthiness was established through including all authors' reflection and engagement in all steps of the analyses. Regarding dependability, the same author conducted the interviews using a semi-structured interview guide. One limitation in this study was the difficulties to engage patients in the interviews for a longer period of time. The interview time ranged between 10 and 24 minutes (mean 15.15). This might be due to the acute phase that patients were in at the time of their interviews and the severity in their illness; however, this was compensated by the inclusion of more participants in the study to have enough data to draw necessary conclusions.

Conclusion

A VR calm room provides a cost-effective, modern, convenient method for relaxation in acute psychiatric care. Patients appreciated the accessibility, convenience and variety of modalities. They also thought that it increased their well-being. VR technology provides potentialities in solving logistic issues and is cost effective. Further research is needed that includes patients in psychiatric inpatient care with a broader range of mental conditions.

For peer review only

Author affiliations

[1] Region Västra Götaland, Sahlgrenska University Hospital, Psychiatric Department, Gothenburg, Sweden

[2]Institute of Health and Care Sciences, Sahlgrenska Academy, University of Gothenburg, Gothenburg, Sweden

[3]Centre for Person-Centred Care (GPCC), Sahlgrenska Academy, University of Gothenburg, Gothenburg, Sweden

[4]Region Västra Götaland, Sahlgrenska University Hospital, Department of Forensic Psychiatry, Gothenburg, Sweden

[5]Institute of Neuroscience and Physiology, University of Gothenburg, Sahlgrenska Academy, Gothenburg, Sweden

[6]Centre for Psychiatry Research, Department of Clinical Neuroscience, Karolinska Institute and Stockholm Health Care Services, Stockholm, Sweden

Acknowledgements We thank the participants who generously shared their experiences in this study. The authors would also like to thank the Psychiatry Affective Department, the managers and, particularly, area manager Mathias Alvidius for their support and assistance in conducting the study. We would also like to thank William Hamilton who developed the virtual reality calm room scenario. We would also like to thank Peter Todd (Tajut Ltd., Kaiapoi, New Zealand) for third-party editorial assistance in drafting of this manuscript, for which he received financial compensation.

Contributors SS, PL, AT and LA were involved in the conceptualization of the study. LA, SW and MI developed the methodology. MI curated the study data. MI, LA

For peer review only

For peer review only - http://bmjopen.bmj.com/site/about/guidelines.xhtml

and SW conducted the formal analysis. MI, LA and SW undertook administration of the project. MI and LA wrote the first draft of the article. All authors were involved in the conduct of the study, review and editing of the article, and approved the submitted version.

Funding The project was funded by Lokala FoU-rådet i Göteborg och Södra Bohuslän with 110,000 Swedish krona and by the University of Gothenburg.

Competing interests PL reports having received minor consulting fees from Mimerse, the company that developed the VR application used. Mimerse is no longer an active company. The other authors declare that they have no competing interests.

Patient consent for publication Not applicable.

Ethics approval Ethical approval was granted by the Swedish Ethical Review Authority (Dnr: 318-18). Written consent was obtained from each participant.

Provenance and peer review Not commissioned; externally peer reviewed.

Data availability statement Due to local regulations, we are not able to share the data freely but raw data can be provided from the corresponding author on reasonable request.

ORCID iDs

For peer review only - http://bmjopen.bmj.com/site/about/guidelines.xhtml

Maria Ilioudi https://orcid.org/0000-0003-0709-7768

Sara Wallström https://orcid.org/0000-0001-7579-4974

Steinn Steingrimsson https://orcid.org/0000-0001-8320-5607

Philip Lindner https://orcid.org/0000-0002-3061-501X

Almira Osmanovic Thunström https://orcid.org/0000-0002-8791-9524

Lilas Ali https://orcid.org/0000-0001-7027-4371

For peer review only

REFERENCES

1 Sandifer PA, Sutton-Grier AE, Ward BP. Exploring connections among nature, biodiversity, ecosystem services, and human health and well-being: opportunities to enhance health and biodiversity conservation. *Ecosystem Services* 2015;12:1–15. https://doi.org/10.1016/j.ecoser.2014.12.007

2 Hedblom M, Gunnarsson B, Iravani B, *et al*. Reduction of physiological stress by urban green space in a multisensory virtual experiment. *Sci Rep* 2019;9:10113. https://doi.org/10.1038/s41598-019-46099-7

3 Klainin-Yobas P, Oo WN, Yew PY, *et al*. Effects of relaxation interventions on depression and anxiety among older adults: a systematic review. *Aging Ment Health* 2015;19:1043–55. https://doi.org/10.1080/13607863.2014.997191

4 Halm MA. Relaxation: a self-care healing modality reduces harmful effects of anxiety. *Am J Crit Care* 2009;18:169-72. https://doi.org/10.4037/ajcc2009867

5 Riches S, Azevedo L, Bird L, *et al*. Virtual reality relaxation for the general population: a systematic review. *Soc Psychiatry Psychiatr Epidemiol* 2021;56:1707–27. https://doi.org/10.1007/s00127-021-02110-z

6 Schrempf MC, Petzold JQ, Vachon H, *et al*. Virtual reality-based relaxation for enhancement of perioperative well-being and quality of life: protocol for a randomised pilot trial. *BMJ Open* 2021;11:e044193. https://doi.org/10.1136/bmjopen-2020-044193

7 Shah LB, Klainin-Yobas P, Torres S, *et al*. Efficacy of psychoeducation and relaxation interventions on stress-related variables in people with mental disorders: a literature review. *Arch Psychiatr Nurs* 2014;28:94–101. https://doi.org/10.1016/j.apnu.2013.11.004

For peer review only

8 Zhang H, Xu H, Zhang Z-X, *et al*. Efficacy of virtual reality-based interventions for patients with breast cancer symptom and rehabilitation management: a systematic review and meta-analysis. *BMJ Open* 2022;12:e051808. https://doi.org/10.1136/bmjopen-2021-051808

9 Li M, Wang L, Jiang M, *et al*. Relaxation techniques for depressive disorders in adults: a systematic review and meta-analysis of randomised controlled trials. *Int J Psychiatry Clin Pract* 2020;24:219–26. https://doi.org/10.1080/13651501.2020.1764587

10 O'Gara G, Murray L, Georgopoulou S, *et al*. SafeSpace: what is the feasibility and acceptability of a codesigned virtual reality intervention, incorporating compassionate mind training, to support people undergoing cancer treatment in a clinical setting?. *BMJ Open* 2022;12:e047626. https://doi.org/10.1136/bmjopen-2020-047626

11 Björkdahl A, Perseius KI, Samuelsson M, *et al*. Sensory rooms in psychiatric inpatient care: staff experiences. *Int J Ment Health Nurs* 2016;25:472–9. https://doi.org/10.1111/inm.12205

12 Iloudi M, Lindner P, Ali L, *et al*. Physical versus virtual reality-based calm rooms for psychiatric inpatients: a quasi-reandomized trial. *JMIR Ment Health* 2023 [in press]

13 Andersen C, Kolmos A, Andersen K, *et al*. Applying sensory modulation to mental health inpatient care to reduce seclusion and restraint: a case control study. *Nord J Psychiatry* 2017;71:525–8. https://doi.org/10.1080/08039488.2017.1346142

14 Cummings KS, Grandfield SA, Coldwell CM. Caring with comfort rooms. Reducing seclusion and restraint use in psychiatric facilities. *J Psychosoc Nurs*

For peer review only

For peer review only - http://bmjopen.bmj.com/site/about/guidelines.xhtml

Ment Health Serv 2010;48:26–30. https://doi.org/10.3928/02793695-20100303-02

15 Novak T, Scanlan J, McCaul D, *et al*. Pilot study of a sensory room in an acute inpatient psychiatric unit. *Australas Psychiatry* 2012;20:401–6. https://doi.org/10.1177/1039856212459585

16 Seckman A, Paun O, Heipp B, *et al*. Evaluation of the use of a sensory room on an adolescent inpatient unit and its impact on restraint and seclusion prevention. *J Child Adolesc Psychiatr Nurs* 2017;30:90–7. https://doi.org/10.1111/jcap.12174

17 Lindner P, Miloff A, Bergman C, *et al*. Gamified, automated virtual reality exposure therapy for fear of spiders: a single-subject trial under simulated real-world conditions. *Front Psychiatry* 2020;11:116. https://doi.org/10.3389/fpsyt.2020.00116

18 Repetto C, Gaggioli A, Pallavicini F, *et al*. (2011). Virtual reality and mobile phones in the treatment of generalized anxiety disorders: a phase-2 clinical trial. *Personal and Ubiquitous Computing* 2011;17:253–60. https://doi.org/10.1007/s00779-011-0467-0

19 Fagernäs S, Hamilton W, Espinoza N, *et al*. What do users think about Virtual Reality relaxation applications? A mixed methods study of online user reviews using natural language processing. *Internet Interv* 2021;24:100370. https://doi.org/10.1016/j.invent.2021.100370

20 Lloyd A, Haraldsdottir E. Virtual reality in hospice: improved patient well-being. *BMJ Support Palliat Care* 2021;11:344–50. https://doi.org/10.1136/bmjspcare-2021-003173

For peer review only - http://bmjopen.bmj.com/site/about/guidelines.xhtml

21 Miloff A, Lindner P, Hamilton W, *et al*. Single-session gamified virtual reality exposure therapy for spider phobia vs. traditional exposure therapy: study protocol for a randomized controlled non-inferiority trial. *Trials* 2016;17:60. https://doi.org/10.1186/s13063-016-1171-1

22 Tarrant J, Viczko J, Cope H. Virtual reality for anxiety reduction demonstrated by quantitative EEG: a pilot study. *Front Psychol* 2018;9:1280. https://doi.org/10.3389/fpsyg.2018.01280

23 Thunström AO, Sarajlic Vukovic I, Ali L, *et al*. Prevalence of virtual reality (VR) games found through mental health categories on STEAM: a first look at VR on commercial platforms as tools for therapy. *Nord J Psychiatry* 2022;76:474–85. https://doi.org/10.1080/08039488.2021.2003859

24 Scarfe P, Glennerster A. The science behind virtual reality displays. *Annu Rev Vis Sci* 2019;5:529–47. https://doi.org/10.1146/annurev-vision-091718-014942

25 Freeman D, Bradley J, Antley A, *et al*. Virtual reality in the treatment of persecutory delusions: randomised controlled experimental study testing how to reduce delusional conviction. *Br J Psychiatry* 2016;209:62–7. https://doi.org/10.1192/bjp.bp.115.176438

26 Lindner P, Dagöö J, Hamilton W, *et al*. Virtual Reality exposure therapy for public speaking anxiety in routine care: a single-subject effectiveness trial. *Cogn Behav Ther* 2021;50:67–87. https://doi.org/10.1080/16506073.2020.1795240

27 Veling W, Lestestuiver B, Jongma M, *et al*. Virtual reality relaxation for patients with a psychiatric disorder: crossover randomized controlled trial. *J Med Internet Res* 2021;23:e17233. https://doi.org/10.2196/17233

For peer review only - http://bmjopen.bmj.com/site/about/guidelines.xhtml

28 Carl E, Stein AT, Levihn-Coon A, *et al*. Virtual reality exposure therapy for anxiety and related disorders: a meta-analysis of randomized controlled trials. *J Anxiety Disord* 2019;61:27–36. https://doi.org/10.1016/j.janxdis.2018.08.003

29 Fodor LA, Coteț CD, Cuijpers P, *et al*. The effectiveness of virtual reality based interventions for symptoms of anxiety and depression: a meta-analysis. *Sci Rep* 2018;8:10323. https://doi.org/10.1038/s41598-018-28113-6

30 Wechsler TF, Kümpers F, Mühlberger A. Inferiority or even superiority of virtual reality exposure therapy in phobias? A systematic review and quantitative meta-analysis on randomized controlled trials specifically comparing the efficacy of virtual reality exposure to gold standard *in vivo* exposure in agoraphobia, specific phobia and social phobia. *Front Psychol* 2019;10:1758. https://doi.org/10.3389/fpsyg.2019.01758

31 Anderson PL, Price M, Edwards SM, *et al*. Virtual reality exposure therapy for social anxiety disorder: a randomized controlled trial. *J Consult Clin Psychol* 2013;81:751–60. https://doi.org/10.1037/a0033559

32 Bouchard S, Dumoulin S, Robillard G, *et al*. Virtual reality compared with *in vivo* exposure in the treatment of social anxiety disorder: a three-arm randomised controlled trial. *Br J Psychiatry* 2017;210:276–83. https://doi.org/10.1192/bjp.bp.116.184234

33 Reger GM, Koenen-Woods P, Zetocha K, *et al*. Randomized controlled trial of prolonged exposure using imaginal exposure vs. virtual reality exposure in active duty soldiers with deployment-related posttraumatic stress disorder (PTSD). *J Consult Clin Psychol* 2016;84:946–59. https://doi.org/10.1037/ccp0000134

For peer review only

34 Botella C, Fernández-Álvarez J, Guillén V, *et al*. Recent progress in virtual reality exposure therapy for phobias: a systematic review. *Curr Psychiatry Rep* 2017;19:42. https://doi.org/10.1007/s11920-017-0788-4

35 Annerstedt M, Jönsson P, Wallergård M, *et al*. Inducing physiological stress recovery with sounds of nature in a virtual reality forest: results from a pilot study. *Physiol Behav* 2013;118:240–50. https://doi.org/10.1016/j.physbeh.2013.05.023

36 Baños RM, Botella C, Guillen V, *et al*. An adaptive display to treat stress-related disorders: EMMA's world. *British Journal of Guidance and Counselling* 2009;37:347–56. https://doi.org/10.1080/03069880902957064

37 Lindner P, Miloff A, Hamilton W, *et al*. The potential of consumer-targeted virtual reality relaxation applications: descriptive usage, uptake and application performance statistics for a first-generation application. *Front Psychol* 2019;10:132. https://doi.org/10.3389/fpsyg.2019.00132

38 Serrano B, Baños RM, Botella C. Virtual reality and stimulation of touch and smell for inducing relaxation: a randomized controlled trial. *Computers in Human Behavior* 2016;55:1–8. https://doi.org/10.1016/j.chb.2015.08.007

39 Parmigiani G, Tortora L, Meynen G, *et al*. Virtual reality interventions for victims of crime: a systematic review. *J Trauma Stress* 2022;35:804–12. https://doi.org/10.1002/jts.22810

40 Dascal J, Reid M, IsHak WW, *et al*. Virtual reality and medical inpatients: a systematic review of randomized, controlled trials. *Innov Clin Neurosci* 2017;14:14–21. PMID: 28386517

41 Pardini S, Gabrielli S, Dianti M, *et al*. The role of personalization in the user experience, preferences and engagement with virtual reality environments for

For peer review only - http://bmjopen.bmj.com/site/about/guidelines.xhtml

relaxation. *Int J Environ Res Public Health* 2022;19:7237. https://doi.org/10.3390/ijerph19127237

42 Krippendorff, K. Content Analysis: an Introduction to its Methodology, 4th ed. Thousand Oaks, CA: Sage Publications 2018.

43 Graneheim UH, Lundman B. Qualitative content analysis in nursing research: concepts, procedures and measures to achieve trustworthiness. *Nurse Educ Today* 2004;24:105–12. https://doi.org/10.1016/j.nedt.2003.10.001

44 Baxter LA. Content analysis. In: Montgomery BM, Duck S, eds., Studying Interpersonal Interaction. The Guilford Press, New York, NY 1991:239–54.

45 Polit DF. Nursing Research: Generating and Assessing Evidence for Nursing Practice, 11th ed. Philadelphia, PA: Wolters Kluwer 2021.

46 World Medical Association. World Medical Association Declaration of Helsinki: ethical principles for medical research involving human subjects. *JAMA* 2013;310:2191–4. doi:10.1001/jama.2013.281053

47 Anderson AP, Mayer MD, Fellows AM, *et al*. Relaxation with immersive natural scenes presented using virtual reality. *Aerosp Med Hum Perform* 2017;88:520–6. https://doi.org/10.3357/amhp.4747.2017

48 Brown P, Waite F, Lambe S, *et al*. Virtual reality cognitive therapy in inpatient psychiatric wards: protocol for a qualitative investigation of staff and patient views across multiple national health service sites. *JMIR Res Protoc* 2020;9:e20300. https://doi.org/10.2196/20300

49 Sanchez-Vives MV, Slater M. From presence to consciousness through virtual reality. *Nat Rev Neurosci* 2005;6:332-339. https://doi.org/10.1038/nrn1651

50 Valmaggia LR, Day F, Rus-Calafell M. Using virtual reality to investigate psychological processes and mechanisms associated with the onset and

For peer review only

maintenance of psychosis: a systematic review. *Soc Psychiatry Psychiatr Epidemiol* 2016;51:921–36. https://doi.org/10.1007/s00127-016-1245-0

51 Browning MH, Mimnaugh KJ, van Riper CJ, *et al*. Can simulated nature support mental health? Comparing short, single-doses of 360-degree nature videos in virtual reality with the outdoors. *Front Psychol* 2020;10:2667. https://doi.org/10.3389/fpsyg.2019.02667

52 Lindner P, Miloff A, Hamilton W, *et al*. Creating state of the art, next-generation Virtual Reality exposure therapies for anxiety disorders using consumer hardware platforms: design considerations and future directions. *Cogn Behav Ther* 2017;46:404–20. https://doi.org/10.1080/16506073.2017.1280843

53 Seabrook E, Kelly R, Foley F, *et al*. Understanding how virtual reality can support mindfulness practice: mixed methods study. *J Med Internet Res* 2020;22:e16106. https://doi.org/10.2196/16106

54 Lindner P, Rozental A, Jurell A, *et al*. Experiences of gamified and automated virtual reality exposure therapy for spider phobia: qualitative study. *JMIR Serious Games* 2020;8:e17807. https://doi.org/10.2196/17807

55 Færden A, Bølgen B, Løvhaug L, *et al*. Patient satisfaction and acute psychiatric inpatient treatment. *Nord J Psychiatry* 2020;74:577–84. https://doi.org/10.1080/08039488.2020.1764620

56 Lindberg MH, Samuelsson M, Perseius KI, *et al*. The experiences of patients in using sensory rooms in psychiatric inpatient care. *Int J Ment Health Nurs* 2019;28:930–9. https://doi.org/10.1111/inm.12593

57 Wykes T, Csipke E, Williams P, *et al*. Improving patient experiences of mental health inpatient care: a randomised controlled trial. *Psychol Med* 2018;48(3):488–97. https://doi.org/10.1017/S003329171700188X

For peer review only - http://bmjopen.bmj.com/site/about/guidelines.xhtml

58 Appel L, Kisonas E, Appel E, *et al*. Administering virtual reality therapy to manage behavioral and psychological symptoms in patients with dementia admitted to an acute care hospital: results of a pilot study. *JMIR Form Res* 2021;5:e22406. https://doi.org/10.2196/22406

For peer review only

FIGURE LEGEND

Figure 1 Screenshots from the virtual reality calm room.

For peer review only

For peer review only - http://bmjopen.bmj.com/site/about/guidelines.xhtml

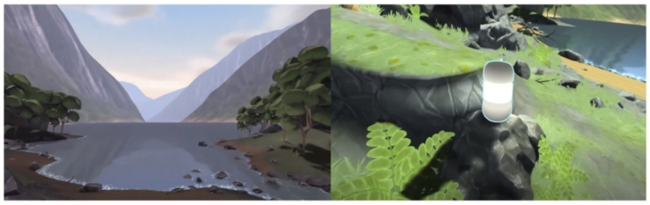

Figure 1

532x753mm (197 x 197 DPI)

COREQ Checklist

Item	Page
1	8
2	1
3	1
4	N/A
5	N/A
6	N/A
7	10
8	8
9	7
10	7
11	7
12	7
13	N/A
14	7
15	7
16	8
17	8
18	N/A
19	9
20	N/A
21	8
22	4, 21
23	N/A
24	9
25	9
26	9
27	N/A
28	N/A
29	12-17
30	12-17
31	10
32	10

For peer review only

BMJ Open

Patient experience of a virtual reality calm room in a psychiatric inpatient care setting in Sweden: a qualitative study with inpatients

Journal:	*BMJ Open*
Manuscript ID	bmjopen-2023-076285.R1
Article Type:	Original research
Date Submitted by the Author:	10-Oct-2023

Complete List of Authors:	Ilioudi, Maria; University of Gothenburg Sahlgrenska Academy, Institute of Health and Care Sciences; Sahlgrenska universitetssjukhuset, Psychiatric Department<br>Wallström, Sara; Institute of Health and Care Sciences, Sahlgrenska Academy, University of Gothenburg; University of Gothenburg Centre for Person-Centred Care, Sahlgrenska Academy, University of Gothenburg<br>Steingrimsson, Steinn; Sahlgrenska universitetssjukhuset, Psychiatric Department; University of Gothenburg Institute of Neuroscience and Physiology, University of Gothenburg<br>Lindner, Philip; Karolinska Institutet, Centre for Psychiatry Research, Department of Clinical Neuroscience<br>Thunström, Almira Osmanovic; University of Gothenburg Institute of Neuroscience and Physiology<br>Ali, Lilas; Health and Care Sciences, Sahlgrenska academy

**Primary Subject Heading**:	Mental health
Secondary Subject Heading:	Health informatics
Keywords:	Virtual Reality, PSYCHIATRY, MENTAL HEALTH

SCHOLARONE™
Manuscripts

I, the Submitting Author has the right to grant and does grant on behalf of all authors of the Work (as defined in the below author licence), an exclusive licence and/or a non-exclusive licence for contributions from authors who are: i) UK Crown employees; ii) where BMJ has agreed a CC-BY licence shall apply, and/or iii) in accordance with the terms applicable for US Federal Government officers or employees acting as part of their official duties; on a worldwide, perpetual, irrevocable, royalty-free basis to BMJ Publishing Group Ltd ("BMJ") its licensees and where the relevant Journal is co-owned by BMJ to the co-owners of the Journal, to publish the Work in this journal and any other BMJ products and to exploit all rights, as set out in our licence.

The Submitting Author accepts and understands that any supply made under these terms is made by BMJ to the Submitting Author unless you are acting as an employee on behalf of your employer or a postgraduate student of an affiliated institution which is paying any applicable article publishing charge ("APC") for Open Access articles. Where the Submitting Author wishes to make the Work available on an Open Access basis (and intends to pay the relevant APC), the terms of reuse of such Open Access shall be governed by a Creative Commons licence – details of these licences and which Creative Commons licence will apply to this Work are set out in our licence referred to above.

Other than as permitted in any relevant BMJ Author's Self Archiving Policies, I confirm this Work has not been accepted for publication elsewhere, is not being considered for publication elsewhere and does not duplicate material already published. I confirm all authors consent to publication of this Work and authorise the granting of this licence.

For peer review only

Patient experience of a virtual reality calm room in a psychiatric inpatient care setting in Sweden: a qualitative study with inpatients

Authors: Maria Ilioudi[1,2], Sara Wallström[1,3,4,5], Steinn Steingrimsson[1,6], Philip Lindner[7], Almira Osmanovic Thunström[1,6], Lilas Ali[1,2,3]

Correspondence to: Assoc. Prof. Steinn Steingrimsson, MD, PhD, Department of Psychiatry, Sahlgrenska University Hospital, Region Västra Götaland, 41650 Gothenburg, Sweden; Tel: 46 313421000: Email: steinn.steingrimsson@neuro.gu.se

Manuscript type: Original research

Abstract length: 287

Text length: 4683

No. of references: 50

No. of graphics: 3 (2 tables/1 figure)

For peer review only

ABSTRACT

Objective Calm rooms have been developed and implemented in psychiatric inpatient care settings to offer patients a dedicated space for relaxation in a convenient and safe environment. Recent technology developments have enabled virtual reality (VR) equivalents of calm rooms that can be feasibly deployed in psychiatric care settings. While research has shown VR environments to be efficacious in inducing relaxation, little is known how these virtual calm rooms are perceived by patients. The aim of this study was to elucidate patient experiences of using a VR calm room in a psychiatric inpatient setting.

Design Qualitative interview study. Semi-structured interviews were analysed using qualitive inductive content analysis, which focuses on the interpretation of texts for making replicable and valid inferences.

Setting Swedish hospital psychiatric inpatient care setting with a wireless, three degrees-of-freedom VR head-mounted display running a calm room application simulating nature environments.

Participants 20 adult patients (12 women) with bipolar disorder (n=18) or unipolar depression (n=2).

Results Participants experienced the use of the VR calm room as having a positive impact on them, inducing awareness, calmness and well-being. They were thankful to be offered a non-pharmacological alternative for anxiety relief. Participants also expressed that they had some concerns, at first, about how they would react emotionally before using the VR device; however, after use, they highlighted that their overall experience was positive. They also expressed that they could see potential for further development of VR technology in psychiatric care.

For peer review only - http://bmjopen.bmj.com/site/about/guidelines.xhtml

Conclusions VR technology has the potential to solve pressing logistic issues in offering calm rooms in psychiatric inpatient care. VR calm rooms appear to be appreciated by psychiatric inpatients, who value their accessibility, convenience and variety of modalities offered. Participants perceived an increase in their well-being after use.

Keywords: virtual reality, psychiatry, relaxation, psychiatric inpatient care, qualitative design, interviews

For peer review only

Strengths and limitations of this study

- This study was conducted in an inpatient psychiatric setting with participants who were introduced to a VR calm room as a new method for relaxation.
- The interview data in this study provided rich descriptions of patient experiences of using the VR calm room.
- There were some difficulties in engaging patients in longer interviews, which was not unexpected given the acute phase that patients were in at that time.
- The short duration of engagement by the informants during interviews resulted in more inclusions (n=20) to have enough data to draw necessary conclusions.
- VR calm room has the potential to be a cost-effective, non-pharmacological form of anxiety relief in inpatient psychiatric setting.

For peer review only

For peer review only - http://bmjopen.bmj.com/site/about/guidelines.xhtml

INTRODUCTION

Patients admitted to psychiatric inpatient wards often experience episodes of heightened emotions, which left uncared for may ultimately necessitate invasive methods such as forced medication, restraints, and one-on-one monitoring, in order to keep them from harming themselves. So called calm rooms (sensory rooms) offer a seemingly attractive alternative to these invasive methods, and consist of multisensory environments that stimulate sensation, olfactory, auditory, vision and, can be decorated with elements such as lights, pictures, iPad, aromatic oils, blankets.[1] The purpose is to offer patients a dedicated space for relaxation, in order to induce well-being through calming exercises and distraction from distress. Guided relaxation and other instructed exercises may also be offered.[2,3,4,5] Calm rooms can also be used to provide self-help through a convenient and safe environment and, at the same time, empower the therapeutic relationship between the patient and healthcare workers at inpatient units.[1,6] Previous research indicates that calm rooms can be used for clinical purposes as a beneficial method for patients in reducing stress and inducing well-being.[1, 4, 5, 7, 8]

Despite well-known benefits, calm rooms are far from ubiquitous in psychiatric inpatient wards, presumably due to the logistic required: there needs to be a dedicated room (with an overall low occupation rate), fitted with special décor and equipment, and special usage procedures are required. Recently, research has begun exploring whether Virtual Reality (VR) technology can be used to create virtual Calm rooms. VR refers computer-generated, perceived three-dimensional and gaze-interactive environments, typically through the use of a head-mounted display.[9] VR technology is increasingly being used in the treatment of mental disorders and numerous interventions have been developed and

For peer review only - http://bmjopen.bmj.com/site/about/guidelines.xhtml

tested.[10,11,12,13,14] The term "virtual reality" has been used to describe a VR environments may promote relaxation through visualisation, engagement and immersion into pleasant virtual environments (often nature simulations), and by removing users from stressful situations.[15]

There are some clinical studies involving VR calm rooms in psychiatric settings which show the efficacy of VR environments for mental health conditions.[8,16,17,18] Moreover, studies have also shown the efficacy [19,20,21] of VR in treating mental conditions, including social anxiety disorder,[22,23] post-traumatic stress disorder,[24] generalised anxiety disorder (GAD) [8] and phobias.[12,25] Furthermore, VR technology has been used to provide immersive experiences for relaxation.[26,27,28,29] A previous study has suggested that VR nature relaxation immediately reduced negative affective states and psychological stress in patients with psychiatric disorders.[18] In addition, Repetto et. al.[8] showed, in a VR pilot study for the treatment of GAD, that using VR as treatment may result in better clinical outcomes at the end of the treatment. The participants also answered that they were very satisfied to use the device in every-day life’s situations to relax.[8]

VR technology solves logistic issues since it can be used anywhere [17, 20] and is affordable in comparison to refurbishing a room at a psychiatric inpatient care setting.[12] The technology has also been shown to be convenient and safe in psychiatric inpatient care settings since it is made of harmless materials and is usually used with supervision until the patient is trusted on his or her own.[30] VR relaxation settings provide a sense of presence, pleasure, activation and engagement, and a personalised experience.[31] However, VR application may not suit everyone or is not appropriate to acute conditions. In addition, the healthcare professionals need training and involving the multidisciplinary team in decision-

For peer review only

making for VR implementation in the ward. Both patients and staff expressed a practical issue about the access to private space to provide the VR application [32]

There is, however, a lack of knowledge of how users in psychiatric inpatients perceive the use of VR-based relaxation technologies. The aim of this study was therefore to elucidate patient experience of using a VR calm room in psychiatric inpatient care.

MATERIAL AND METHODS

Study design

This study has a qualitative and descriptive design. Semi-structured interviews were conducted and analysed using a qualitive content analysis based on inductive theory. This study was part of a larger research project aimed at evaluating the effect of VR and physical calm rooms in psychiatric inpatient care.[2]

Setting and participants

Inclusion criteria were age ≥18 years, admitted for psychiatric inpatient care at a care setting specialised in bipolar disorder in the western region of Sweden (Sahlgrenska University Hospital, Gothenburg) and having previously used the VR calm room. Due to a lack of empty beds in other settings, patients with other psychiatric diagnoses were also included in this study. A total of 25 patients fulfilled the inclusion criteria and were asked to participate in the study by the first author. Of those, 5 declined and 20 agreed to an interview. There was no overlap in samples between the current study and a previously reported trial.[2]

For peer review only

For peer review only - http://bmjopen.bmj.com/site/about/guidelines.xhtml

Procedure

Patients who used the VR calm room received instructions on the VR headset's functions and how to navigate in the virtual environment. To engage with the device, patients used a wireless, three degrees-of-freedom VR head-mounted display (an Oculus Go) running the Calm Room application developed by Mimerse. Example screenshots of the environment are shown in figure 1. The application offers a selection of different soothing nature environments (e.g. a desert, a forest beach) that can be tailored through, for example, the day and night cycle and dynamic weather. In addition, users may engage in breathing exercises, mindfulness programs and relaxing music. The user can interact with the environment by looking at different objects inside the program and using the handheld controller to adjust preferences and build individualised scenery. The interactive smorgasbord approach was chosen in light of research showing that users have different preferences for different virtual nature environments [33], as well as non-VR research showing the importance of matching soothing music to user preferences.[34]

Data collection

The individual interviews all followed a semi-structured interview-guide developed by the authors. The interviews were conducted between October 2021 and May 2022 by the first author (MI). Interviews took place at the psychiatric inpatient care setting during patients' care time. The mean duration of the interviews was 15.15 (range, 10–24) minutes. The interviews were recorded and transcribed verbatim. The participants were asked, for example, how they experienced the calm room, was there anything that worried them when using the VR calm room, how do they think an alternative to the VR calm room can be developed and what kind of difference do

For peer review only - http://bmjopen.bmj.com/site/about/guidelines.xhtml

they think using the VR calm room has made for their situation. After the conduction of 20 interviews, data saturation was attained.

Patient and public involvement

Patients were involved in the design of the VR environment.

Data analysis

Data were analysed using qualitative inductive content analysis, which search for patterns in the text, aiming to acquire a theoretical understanding from collected interview data.[35] The inductive approach entails that text is analysed based on the participants' narratives through grouping meaning units containing aspects related to each other in content and context.[36] Baxter [37] defines 'meaning unit' as the constellation of words or statements that relate to the same central meaning by achieving a condensed description of the phenomenon by emphasising differences and similarities within the data. A theme is a thread of an underlying meaning through meaning units or codes on an interpretive level. Meaning-units were identified according to the study aim, which were then condensed and labelled with codes. [37]

First, the first and last authors (MI and LA) familiarised themselves with the material by reading the transcriptions several times in order to grasp the central meaning of the texts. Through careful reading, relevant sentences and phrases to the study aim were identified. These were then used in the constellation of meaning units and to begin the coding process. The codes were then sorted and abstracted into themes and subthemes. To ensure trustworthiness of the themes, the first and

For peer review only - http://bmjopen.bmj.com/site/about/guidelines.xhtml

last author worked closely with the analysis. The other authors validated the text and discussed the themes and subthemes until consensus was reached.

Ethical considerations

Ethical approval was granted by the Swedish Ethical Review Authority (Dnr: 318-18). All participants received verbal and written information about the study and that they could end the interview at any time without specifying a reason. All data were treated confidentially. All ethical decisions were guided by the Declaration of Helsinki [38] and its subsequent amendments The participants admitted to the psychiatric inpatient care setting may have been under compulsory psychiatry care. The authors were aware that the participants' mental health condition might be affected and were prepared to interrupt the research process or offer suitable support services upon participant request or if this was deemed necessary.

RESULTS

A total of 20 patients (12 women and 8 men) participated in the study. Participant characteristics are summarised in table 1.

Table 1 Patient demographic and clinical characteristics (n=20)

Variable	No. of patients
Sex	
Men	8
Women	12
Age, years	
20–29	4
30–49	5
50–59	5
60–69	6
Diagnosis	
Bipolar disorder	18
Unipolar depression	2

For peer review only - http://bmjopen.bmj.com/site/about/guidelines.xhtml

The first overall impression of the analysis revealed that using the VR calm room had a positive impact on the participants during their period of care at the inpatient psychiatric unit. The experience raised feelings of awareness, calmness and well-being. Participants also had suggestions on further development of the VR technology in mental healthcare in the future. An overview of the themes and sub-themes is presented in table 2.

Table 2 Themes and sub-themes from interviews following VR calm room use

**Feelings before and during use**	**Negative point of view**	**Potential for future development**
Being offered an alternative to medication	Practical issues and side effects	Willing to continue use
Feelings of relief and relaxation	Unpleasant experiences	Recommending VR
Sense of independency		

For peer review only

Feelings before and during use

Participants expressed that they had a positive attitude about using the calm room before the intervention and thought it would be an enjoyable part in the overall time of care at the psychiatric inpatient unit. Several participants mentioned that they preferred non-pharmacological alternatives such as VR. Furthermore, they experienced a sense of independence and gave positive feedback about the opportunity to learn new relaxation methods.

Being offered an alternative to medication

During inpatient psychiatric care, the participants sometimes encountered boundaries in being able to achieve a peaceful state of mind on their own due to the care environment being hectic or perceived as stressful. They perceived there to be few non-pharmacological alternatives available or any support offered to them to enhance their well-being and reduce anxiety. They therefore expressed gratitude over being offered the use the VR device as an alternative to drugs.

The participants also mentioned that use of the virtual calm room was very anxiety-relieving and therefore had a positive impact on their well-being during the time they were admitted to the inpatient psychiatry unit. Being admitted to psychiatric inpatient care was typically associated with high levels of anxiety and feelings of seclusion. The participants highlighted that inpatient psychiatric care needs alternative methods that they can be offered for anxiety reduction and self-support. The participants also stated that they were upset about the limited options as well as the traditional relaxation methods previously available and therefore really appreciated the time that they spent in the VR calm room. The VR calm room enriched their day at the psychiatric inpatient care setting by giving them a new

For peer review only - http://bmjopen.bmj.com/site/about/guidelines.xhtml

dimension to rely on when needed. The participants described the VR rooms as a strategy with which they could find respite and develop emotional self-care in a stressful environment at the psychiatric inpatient care setting. Participants also highlighted that the VR represented something innovative in psychiatric care which gives hope for new treatment options.

> "…one thing is that we have a lot of time here, it's slow, you have nothing to do, you maybe feel bad, if it's anxiety. That you get a tool and you sit for a certain time. I think that can help, because sometimes you might just go and go and go and do nothing, then it doesn't get better, there are no…you have to have multiple tools. This…virtual room, well, I think it's a good thing..." P1

Feelings of relief and relaxation

Participants had varying experiences of how VR could potentially influence their mental condition in a better way. The experiences of use were described as being helpful for their condition and that it gave them a sense of a holistic approach to their feelings. They expressed feelings of joy and that it was something beautiful to experience. Participants also stated that using the VR calm room fulfilled their day because they felt that they had done something creative and worthwhile for their condition. They described the environment they experienced in the VR device as dynamic and that they found some kind of a new dimension to strategically enhance their well-being. Participants also experienced the VR environment to induce a state of calm and creating an awareness of their emotions at the time of use. They found it comfortable to go into another reality and leave the physical one behind for a while.

For peer review only - http://bmjopen.bmj.com/site/about/guidelines.xhtml

Participants in this study usually experienced a stressful reality on a daily basis which contributed to their feelings of anxiety.

> "...it felt very nice to enter another world and then that I felt calmer afterwards…" P1

Sense of independency

The VR device was viewed as an opportunity for participants to learn new relaxation methods, such as relaxing the body, practicing breathing and maintaining positive thinking. Participants expressed that it was easy to learn and practice guided meditation using the VR application. Furthermore, they mentioned that VR is easier to handle compared to usual relaxation methods that they would use in the "real world". Participants wished to stay longer in the VR calm room because they described their use as a new way to reach a feeling of calmness. Participants also expressed that using the VR room gave them a sense of independence in the psychiatric inpatient care setting by allowing them to make their own decisions and becoming more involved in their own care.

> "…It's not easy to describe. Now I'll see, you first came...completely shocked by that nature and then feelings just grow, it's nice inside you that fills up the soul…" P7

Participants indicated that the device was comfortable and flexible to use. They could, on their own, decide how they wanted their calm room to be and the duration of the session. This was all handled by themselves, which gave them a sense of

For peer review only

independence in the psychiatric inpatient unit. Furthermore, participants expressed that being able to stay in the room at the psychiatric inpatient care setting solved logistic issues and could therefore be used at any place in the ward based on their own individual preferences. They also appreciated that they could pause, end and restart the session at any time.

> "…If you're alone, if no staff is here, the VR glasses would have been good because then you can also lie down. I thought that was good. In this relaxation thing, they don't nag. In some relaxation videos it's, tie the hand, open the hand, tie, open. Here it was just, tie the hand and then you did the rest yourself. So that was enough information…" P5

Negative point of view

This theme describes the participants' negative experiences and feelings related to the use of the VR device. Some experienced unpleasant feelings at the start when first using the VR calm room. They were afraid and worried about some components of the VR nature environment such as the darkness and birds. Some of the participants also mentioned that the device was not very comfortable to wear on their head.

Practical issues and side effects

Participants experienced negative issues with the VR device being uncomfortable and heavy to carry for a longer period of time on their head. Some participants mentioned that the VR device did not fit properly and would therefore slip during the time they used it. This affected the overall experience of VR device use. They also

For peer review only - http://bmjopen.bmj.com/site/about/guidelines.xhtml

mentioned that sometimes they would become dizzy and feel nauseous while using the device. Participants also mentioned that these issues usually came at the start of the session and would become less common as they became more familiar with the VR environment. None of the participants interrupted their session due to cybersickness. Before using the VR device, they had their hesitations, some of them worried that they might feel a bit dizzy and uncomfortable. However, they managed to overcome that, after using the VR device for a while, finding it was not as frightening or strange as they thought that it would be. The participants expressed that, when they used the VR device, they felt more relaxed at the end of the session than before.

"...but that it slipped and then it got on the glasses and then there was no sharpness so I had to take it up all the time..." P3

Unpleasant experiences

Entering the VR world was for some participants an unpleasant experience because they were sometimes stressed and worried for no reason that they can recall. They were also expecting their own imagination to kick in and that is why they did not want to be left alone in the VR calm room. They indicated that they were waiting for more life and interaction in the virtual world such as with animals or other creatures.

"…At first it was a bit strange and scary because when I turned my head there was like nothing but the VR environment and then I got stressed at first because I felt it was too close and I didn't recognise myself. But then it got better and then it

For peer review only - http://bmjopen.bmj.com/site/about/guidelines.xhtml

became a bit like my environment, my own. So it turned around and got better..." P5

Furthermore, the analysis indicated that some of the participants experienced VR as frightening and an unsafe environment when specific images would appear such as birds flying everywhere or something else unexpected that might happen in the VR environment making them stay alert in case something unexpected would occur. Participants also described that one scenario in the VR was too dark, which usually led to them becoming anxious of the light and therefore interpreting the experience as being uncomfortable as it would remind them of walking into death when you see the light at the end of the session.

"...I perceived the light as...a bit like death, that you walk towards the light, that it became too much...that it became too peaceful..." P4

"…Then the birds, there were so many birds, it didn't feel like a threat but it felt a bit stressful, I don't like seagulls, so it got a bit like this..." P15

Potential for future development

This theme captures participants' suggestions and recommendation on future use of a VR calm room and what kind of potentials they see for further development, for example, VR as an alternative to treatment for stress reduction.

Willing to continue use

For peer review only

The VR experience was described as a new experience for the participants. They stated that they would consider using the VR device again both in a healthcare setting and also by themselves for self-care at home. They wanted to have easy accessibility to the VR device every day in the psychiatric inpatient care setting and wished that there would be several VR devices available for use. Furthermore, the participants expressed that they wished to use VR on a daily basis as a part of their treatment. Several participants wanted to have a VR device of their own and asked if they could buy one or get access to the calm room application. The main statement that was given by the participants in this study was that they wanted to use the device again in the future.

"…I want to use it again and I was still sceptical before, I was open-minded, sceptical, but I would like to use it again if I get the chance..." P20

Recommending VR

Participants mentioned that VR could reduce anxiety and induce well-being. They recommended the use of the VR device for therapeutic purposes as they could see potential for that. Despite some frightening features, the participants also described use as a positive and not harmful experience, which is why they would recommend it to others.

"…It's relaxing so it affects everyone positively, it can be positive for everyone..." P7

For peer review only - http://bmjopen.bmj.com/site/about/guidelines.xhtml

However, there were some participants who mentioned that they thought that the VR concept might be inappropriate for patients who have vision problems and difficulties with crisis situations, such as for some patients with psychosis. It might also be inappropriate to use when feeling too tired or trapped. Participants wished for further functions to be developed in the VR device and also wanted to be able to choose different environments, not just the natural calm room that was programmed. They wanted to experience more dynamics, and visions that included more animals and other creatures than what they had experienced. The participants recommended and wished to use the VR for about 15 to 20 minutes as they felt that in that time they could experience the full effect of anxiety relief.

DISCUSSION

The aim of this study was to elucidate experiences of using a VR calm room by patients admitted to psychiatric inpatient care. Our findings strengthen the results of previous research, which showed VR calm rooms to increase well-being and lower distress. [26,28,39] In the current study, participants expressed feelings of relaxation, which induced their well-being, when using the VR room. Implementation of VR technology in psychiatric inpatient care settings has been shown to successfully improve patient recovery and emotional self-care [40] as a VR calm room strengthens patient choices, leading to a flexible and convenient strategy for resolving distress in an acute psychiatric setting. [2] Such methods might enhance patient engagement in their care and to take on the responsibility for recovery of their severe mental condition.

The findings support that the VR calm room reduces the experience of stress and helped patients to feel better with a sense of calm. Previous studies have shown that

For peer review only

immersion into VR environments has positive psychological and physical effects.[17, 41] Previous research has found that a VR calm room design can distract patients from stressful acute psychiatric settings.[42] The design of the VR room in our study, which aimed at creating a less hospital-like experience with features supporting familiarity and flexibility, may have contributed to the patients' sense of safety, self-control and enjoyment. Exposure to VR nature images can provide emotional well-being for patients who do not have access to outdoor activities and nature.[43] Patients stay in psychiatric inpatient care for long periods of time and VR calm rooms have the potential to offer visual access to nature and its positive effects. VR technology may solve logistics issues in the psychiatric inpatient care as patients can use the VR device in their own room in the psychiatric inpatient setting without affecting safety.[10,44] In comparison to traditional relaxation methods, participants mentioned that the VR calm room was easy to access and use. VR relaxation is appreciated by users,[45] and has become affordable and more accessible, which has led to its increasingly widespread use in psychiatric care.[42]

Participants reported several negative experiences with the use of VR. Some patients expressed that the experience was unpleasant and them being worried for no reason. They also mentioned becoming dizzy and that some of the images in the VR calm room were scary. The unpleasant experiences might be due to the new environment and unfamiliar components. Our findings align with previous studies where users reported issues with the installation process, overheating, the visual presentation and negative effects such as anxiety.[10] It is possible that some of these negative experiences may be due to personal preferences, which might be avoidable by offering the user a variety of environments.[46] Furthermore, patients admitted to psychiatric inpatient care experience low satisfaction with their influence

For peer review only

For peer review only - http://bmjopen.bmj.com/site/about/guidelines.xhtml

on the choice of treatment and medication.[47] Non-pharmacological methods such as physical calm rooms have safety issues; therefore, healthcare workers may become risk averse and initially worried about letting patients stay on their own in calm rooms. [48] However, activities that break up the monotony in psychiatric inpatient care settings were appreciated by the patients.[49]

The participants in this study highlighted some future recommendations and suggestions about VR calm rooms such as creation of several scenarios and device options with more modalities. This is in line with previous research which suggests that the VR scenario should be expanded to include various additional experiences such as live music scenes, scenes featuring people walking around and animals.[50] Patients recommended the use of the VR device as a therapeutic method for relaxation and stress reduction both in healthcare settings and for patients to use on their own at home. However, some participants mentioned that VR might be inappropriate for patients with vision problems and when being too tired. Also, some recommended avoiding the use of VR in crisis psychiatric conditions due to the high levels of anxiety and lower adaptation ability in these circumstances. Self-relaxation is an important feature of the care process in psychiatric inpatient care; therefore, participants stated that they wanted to use the VR room in the future as a relaxation tool. Broader use of VR calm rooms should be investigated to explore their potential for being part of usual treatment in acute psychiatric care with individualized content. Important considerations in inpatient setting are agitation and risk for violence, which have to be evaluated in each case. Presumably, a patient that is used to the technique might benefit in an agitated state but studies on the safety of such an intervention are needed.

For peer review only - http://bmjopen.bmj.com/site/about/guidelines.xhtml

Study strengths and limitations

The primary strength of this study is that it was conducted in inpatient psychiatric care, with fully representative patients. It is especially important to include patients who are diagnosed with severe acute psychiatric conditions as they are often excluded from studies due to their illness. This study had a sample of participants with bipolar diagnosis and unipolar depression. The interview data in this study provided rich descriptions of patient experiences of using the VR calm room. Moreover, we reached a broad population sample by interviewing women and men of different ages. The qualitative approach using content analysis [35,36,37] made it possible to elucidate patient experiences using a VR calm room in acute psychiatric care. Trustworthiness was established through including all authors' reflection and engagement in all steps of the analyses. Regarding dependability, the same author conducted the interviews using a semi-structured interview guide. One limitation in this study was the difficulties to engage patients in the interviews for a longer period of time. The interview time ranged between 10 and 24 minutes (mean 15.15). This might be due to the acute phase that patients were in at the time of their interviews and the severity in their illness; however, this was compensated by the inclusion of more participants in the study to have enough data to draw necessary conclusions.

Conclusion

A VR calm room provides a cost-effective, modern, convenient method for relaxation in acute psychiatric care. Patients appreciated the accessibility, convenience and variety of modalities. They also thought that it increased their well-being. VR

For peer review only - http://bmjopen.bmj.com/site/about/guidelines.xhtml

technology provides potentialities in solving logistic issues and is cost effective. Further research is needed that includes patients in psychiatric inpatient care with a broader range of mental conditions.

For peer review only

Author affiliations

[1] Region Västra Götaland, Sahlgrenska University Hospital, Psychiatric Department, Gothenburg, Sweden

[2]Institute of Health and Care Sciences, Sahlgrenska Academy, University of Gothenburg, Gothenburg, Sweden

[3]Centre for Person-Centred Care (GPCC), Sahlgrenska Academy, University of Gothenburg, Gothenburg, Sweden

[4]Region Västra Götaland, Sahlgrenska University Hospital, Department of Forensic Psychiatry, Gothenburg, Sweden

[5]Centre for Ethics, Law and Mental Health (CELAM), University of Gothenburg, Gothenburg, Sweden

[6]Institute of Neuroscience and Physiology, University of Gothenburg, Sahlgrenska Academy, Gothenburg, Sweden

[7]Centre for Psychiatry Research, Department of Clinical Neuroscience, Karolinska Institute and Stockholm Health Care Services, Stockholm, Sweden

Acknowledgements We thank the participants who generously shared their experiences in this study. The authors would also like to thank the Psychiatry Affective Department, the managers and, particularly, area manager Mathias Alvidius for their support and assistance in conducting the study. We would also like to thank William Hamilton who developed the virtual reality calm room scenario. We would also like to thank Peter Todd (Tajut Ltd., Kaiapoi, New Zealand) for third-party editorial assistance in drafting of this manuscript, for which he received financial compensation.

For peer review only - http://bmjopen.bmj.com/site/about/guidelines.xhtml

Contributors SS, PL, AT and LA were involved in the conceptualization of the study. LA, SW and MI developed the methodology. MI curated the study data. MI, LA and SW conducted the formal analysis. MI, LA and SW undertook administration of the project. MI and LA wrote the first draft of the article. All authors were involved in the conduct of the study, review and editing of the article, and approved the submitted version.

Funding The project was funded by Lokala FoU-rådet i Göteborg och Södra Bohuslän with 110,000 Swedish krona.SS received funding from The Söderström König Foundation (SLS-974247).

Competing interests PL reports having received minor consulting fees from Mimerse, the company that developed the VR application used. Mimerse is no longer an active company. The other authors declare that they have no competing interests.

Patient consent for publication Not applicable.

Ethics approval Ethical approval was granted by the Swedish Ethical Review Authority (Dnr: 318-18). Written consent was obtained from each participant.

Provenance and peer review Not commissioned, externally peer reviewed.

For peer review only

Data availability statement Due to local regulations, we are not able to share the data freely but raw data can be provided from the corresponding author on reasonable request.

ORCID iDs

Maria Ilioudi https://orcid.org/0000-0003-0709-7768

Sara Wallström https://orcid.org/0000-0001-7579-4974

Steinn Steingrimsson https://orcid.org/0000-0001-8320-5607

Philip Lindner https://orcid.org/0000-0002-3061-501X

Almira Osmanovic Thunström https://orcid.org/0000-0002-8791-9524

Lilas Ali https://orcid.org/0000-0001-7027-4371

For peer review only

REFERENCES

1 Björkdahl A, Perseius KI, Samuelsson M, *et al*. Sensory rooms in psychiatric inpatient care: staff experiences. *Int J Ment Health Nurs* 2016;25:472–9. https://doi.org/10.1111/inm.12205

2 Ilioudi M, Lindner P, Ali L, et al. Physical Versus Virtual Reality–Based Calm Rooms for Psychiatric Inpatients: Quasi-Randomized Trial. *Journal of Medical Internet Research 2023; 25*. https://doi.org/10.2196/42365

3 Andersen C, Kolmos A, Andersen K, *et al*. Applying sensory modulation to mental health inpatient care to reduce seclusion and restraint: a case control study. *Nord J Psychiatry* 2017;71:525–8. https://doi.org/10.1080/08039488.2017.1346142

4 Cummings KS, Grandfield SA, Coldwell CM. Caring with comfort rooms. Reducing seclusion and restraint use in psychiatric facilities. *J Psychosoc Nurs Ment Health Serv* 2010;48:26–30. https://doi.org/10.3928/02793695-20100303-02

5 Novak T, Scanlan J, McCaul D, *et al*. Pilot study of a sensory room in an acute inpatient psychiatric unit. *Australas Psychiatry* 2012;20:401–6. https://doi.org/10.1177/1039856212459585

6 Seckman A, Paun O, Heipp B, *et al*. Evaluation of the use of a sensory room on an adolescent inpatient unit and its impact on restraint and seclusion prevention. *J Child Adolesc Psychiatr Nurs* 2017;30:90–7. https://doi.org/10.1111/jcap.12174

For peer review only

7 Lindner P, Miloff A, Bergman C, *et al*. Gamified, automated virtual reality exposure therapy for fear of spiders: a single-subject trial under simulated real-world conditions. *Front Psychiatry* 2020;11:116. https://doi.org/10.3389/fpsyt.2020.00116

8 Repetto C, Gaggioli A, Pallavicini F, *et al*. (2011). Virtual reality and mobile phones in the treatment of generalized anxiety disorders: a phase-2 clinical trial. *Personal and Ubiquitous Computing* 2011;17:253–60. https://doi.org/10.1007/s00779-011-0467-0

9 Scarfe P, Glennerster A. The science behind virtual reality displays. *Annu Rev Vis Sci* 2019;5:529–47. https://doi.org/10.1146/annurev-vision-091718-014942

10 Fagernäs S, Hamilton W, Espinoza N, *et al*. What do users think about Virtual Reality relaxation applications? A mixed methods study of online user reviews using natural language processing. *Internet Interv* 2021;24:100370. https://doi.org/10.1016/j.invent.2021.100370

11 Lloyd A, Haraldsdottir E. Virtual reality in hospice: improved patient well-being. *BMJ Support Palliat Care* 2021;11:344–50. https://doi.org/10.1136/bmjspcare-2021-003173

12 Miloff A, Lindner P, Hamilton W, *et al*. Single-session gamified virtual reality exposure therapy for spider phobia vs. traditional exposure therapy: study protocol for a randomized controlled non-inferiority trial. *Trials* 2016;17:60. https://doi.org/10.1186/s13063-016-1171-1

13 Tarrant J, Viczko J, Cope H. Virtual reality for anxiety reduction demonstrated by quantitative EEG: a pilot study. *Front Psychol* 2018;9:1280. https://doi.org/10.3389/fpsyg.2018.01280

For peer review only

For peer review only - http://bmjopen.bmj.com/site/about/guidelines.xhtml

14 Thunström AO, Sarajlic Vukovic I, Ali L, *et al*. Prevalence of virtual reality (VR) games found through mental health categories on STEAM: a first look at VR on commercial platforms as tools for therapy. *Nord J Psychiatry* 2022;76:474–85. https://doi.org/10.1080/08039488.2021.2003859

15 Riches S, Azevedo L, Bird L, *et al*. Virtual reality relaxation for the general population: a systematic review. *Soc Psychiatry Psychiatr Epidemiol*

16 Freeman D, Bradley J, Antley A, *et al*. Virtual reality in the treatment of persecutory delusions: randomised controlled experimental study testing how to reduce delusional conviction. *Br J Psychiatry* 2016;209:62–7. https://doi.org/10.1192/bjp.bp.115.176438

17 Lindner P, Dagöö J, Hamilton W, *et al*. Virtual Reality exposure therapy for public speaking anxiety in routine care: a single-subject effectiveness trial. *Cogn Behav Ther* 2021;50:67–87. https://doi.org/10.1080/16506073.2020.1795240

18 Veling W, Lestestuiver B, Jongma M, *et al*. Virtual reality relaxation for patients with a psychiatric disorder: crossover randomized controlled trial. *J Med Internet Res* 2021;23:e17233. https://doi.org/10.2196/17233

19 Carl E, Stein AT, Levihn-Coon A, *et al*. Virtual reality exposure therapy for anxiety and related disorders: a meta-analysis of randomized controlled trials. *J Anxiety Disord* 2019;61:27–36. https://doi.org/10.1016/j.janxdis.2018.08.003

20 Fodor LA, Coteț CD, Cuijpers P, *et al*. The effectiveness of virtual reality based interventions for symptoms of anxiety and depression: a meta-analysis. *Sci Rep* 2018;8:10323. https://doi.org/10.1038/s41598-018-28113-6

21 Wechsler TF, Kümpers F, Mühlberger A. Inferiority or even superiority of virtual reality exposure therapy in phobias? A systematic review and quantitative meta-analysis on randomized controlled trials specifically comparing the efficacy of

For peer review only - http://bmjopen.bmj.com/site/about/guidelines.xhtml

virtual reality exposure to gold standard *in vivo* exposure in agoraphobia, specific phobia and social phobia. *Front Psychol* 2019;10:1758. https://doi.org/10.3389/fpsyg.2019.01758

22 Anderson PL, Price M, Edwards SM, *et al*. Virtual reality exposure therapy for social anxiety disorder: a randomized controlled trial. *J Consult Clin Psychol* 2013;81:751–60. https://doi.org/10.1037/a0033559

23 Bouchard S, Dumoulin S, Robillard G, *et al*. Virtual reality compared with *in vivo* exposure in the treatment of social anxiety disorder: a three-arm randomised controlled trial. *Br J Psychiatry* 2017;210:276–83. https://doi.org/10.1192/bjp.bp.116.184234

24 Reger GM, Koenen-Woods P, Zetocha K, *et al*. Randomized controlled trial of prolonged exposure using imaginal exposure vs. virtual reality exposure in active duty soldiers with deployment-related posttraumatic stress disorder (PTSD). *J Consult Clin Psychol* 2016;84:946–59. https://doi.org/10.1037/ccp0000134

25 Botella C, Fernández-Álvarez J, Guillén V, *et al*. Recent progress in virtual reality exposure therapy for phobias: a systematic review. *Curr Psychiatry Rep* 2017;19:42. https://doi.org/10.1007/s11920-017-0788-4

26 Annerstedt M, Jönsson P, Wallergård M, *et al*. Inducing physiological stress recovery with sounds of nature in a virtual reality forest: results from a pilot study. *Physiol Behav* 2013;118:240–50. https://doi.org/10.1016/j.physbeh.2013.05.023

27 Baños RM, Botella C, Guillen V, *et al*. An adaptive display to treat stress-related disorders: EMMA's world. *British Journal of Guidance and Counselling* 2009;37:347–56. https://doi.org/10.1080/0306988090297064

For peer review only

28 Lindner P, Miloff A, Hamilton W, *et al*. The potential of consumer-targeted virtual reality relaxation applications: descriptive usage, uptake and application performance statistics for a first-generation application. *Front Psychol* 2019;10:132. https://doi.org/10.3389/fpsyg.2019.00132

29 Serrano B, Baños RM, Botella C. Virtual reality and stimulation of touch and smell for inducing relaxation: a randomized controlled trial. *Computers in Human Behavior* 2016;55:1–8. https://doi.org/10.1016/j.chb.2015.08.007

30 Dascal J, Reid M, IsHak WW, *et al*. Virtual reality and medical inpatients: a systematic review of randomized, controlled trials. *Innov Clin Neurosci* 2017;14:14–21. PMID: 28386517

31 Pardini S, Gabrielli S, Dianti M, *et al*. The role of personalization in the user experience, preferences and engagement with virtual reality environments for relaxation. *Int J Environ Res Public Health* 2022;19:7237. https://doi.org/10.3390/ijerph19127237

32 Brown P, Waite F, Lambe S, *et. al*. Automated Virtual Reality Cognitive Therapy (gameChange) in Inpatient Psychiatric Wards: Qualitative Study of Staff and Patient Views Using an Implementation Framework. *JMIR Formative Research* 2022; *6*:34225. https://doi.org/10.2196/34225

33 Gao T, Zhang T, Zhu L, *et. al*.. Exploring psychophysiological restoration and individual preference in the different environments based on virtual reality. *International Journal of Environmental Research and Public Health* 2019; *16*:3102 https://doi.org/10.3390/ijerph16173102

34 Jiang J, Zhou L, Rickson D, *et al.* (2013). The effects of sedative and stimulative music on stress reduction depend on music preference. *The Arts in Psychotherapy*, 2013:40; 201-205. https://doi.org/10.1016/j.aip.2013.02.002

35 Krippendorff, K. Content Analysis: an Introduction to its Methodology, 4th ed. Thousand Oaks, CA: Sage Publications 2018.

36 Graneheim UH, Lundman B. Qualitative content analysis in nursing research: concepts, procedures and measures to achieve trustworthiness. *Nurse Educ Today* 2004;24:105–12. https://doi.org/10.1016/j.nedt.2003.10.001

37 Baxter LA. Content analysis. In: Montgomery BM, Duck S, eds., Studying Interpersonal Interaction. The Guilford Press, New York, NY 1991:239–54.

38 World Medical Association. World Medical Association Declaration of Helsinki: ethical principles for medical research involving human subjects. *JAMA* 2013;310:2191–4. doi:10.1001/jama.2013.281053

39 Anderson AP, Mayer MD, Fellows AM, *et al*. Relaxation with immersive natural scenes presented using virtual reality. *Aerosp Med Hum Perform* 2017;88:520–6. https://doi.org/10.3357/amhp.4747.2017

40 Brown P, Waite F, Lambe S, *et al*. Virtual reality cognitive therapy in inpatient psychiatric wards: protocol for a qualitative investigation of staff and patient views across multiple national health service sites. *JMIR Res Protoc* 2020;9:e20300. https://doi.org/10.2196/20300

41 Sanchez-Vives MV, Slater M. From presence to consciousness through virtual reality. *Nat Rev Neurosci* 2005;6:332-339. https://doi.org/10.1038/nrn1651

42 Valmaggia LR, Day F, Rus-Calafell M. Using virtual reality to investigate psychological processes and mechanisms associated with the onset and

For peer review only

maintenance of psychosis: a systematic review. *Soc Psychiatry Psychiatr Epidemiol* 2016;51:921–36. https://doi.org/10.1007/s00127-016-1245-0

43 Browning MH, Mimnaugh KJ, van Riper CJ, *et al*. Can simulated nature support mental health? Comparing short, single-doses of 360-degree nature videos in virtual reality with the outdoors. *Front Psychol* 2020;10:2667. https://doi.org/10.3389/fpsyg.2019.02667

44 Lindner P, Miloff A, Hamilton W, *et al*. Creating state of the art, next-generation Virtual Reality exposure therapies for anxiety disorders using consumer hardware platforms: design considerations and future directions. *Cogn Behav Ther* 2017;46:404–20. https://doi.org/10.1080/16506073.2017.1280843

45 Seabrook E, Kelly R, Foley F, *et al*. Understanding how virtual reality can support mindfulness practice: mixed methods study. *J Med Internet Res* 2020;22:e16106. https://doi.org/10.2196/16106

46 Lindner P, Rozental A, Jurell A, *et al*. Experiences of gamified and automated virtual reality exposure therapy for spider phobia: qualitative study. *JMIR Serious Games* 2020;8:e17807. https://doi.org/10.2196/17807

47 Færden A, Bølgen B, Løvhaug L, *et al*. Patient satisfaction and acute psychiatric inpatient treatment. *Nord J Psychiatry* 2020;74:577–84. https://doi.org/10.1080/08039488.2020.1764620

48 Lindberg MH, Samuelsson M, Perseius KI, *et al*. The experiences of patients in using sensory rooms in psychiatric inpatient care. *Int J Ment Health Nurs* 2019;28:930–9. https://doi.org/10.1111/inm.12593

49 Wykes T, Csipke E, Williams P, *et al*. Improving patient experiences of mental health inpatient care: a randomised controlled trial. *Psychol Med* 2018;48(3):488–97. https://doi.org/10.1017/S003329171700188X

For peer review only

50 Appel L, Kisonas E, Appel E, *et al*. Administering virtual reality therapy to manage behavioral and psychological symptoms in patients with dementia admitted to an acute care hospital: results of a pilot study. *JMIR Form Res* 2021;5:e22406. https://doi.org/10.2196/22406

FIGURE LEGEND

Figure 1 Screenshots from the virtual reality calm room.

For peer review only

For peer review only

For peer review only - http://bmjopen.bmj.com/site/about/guidelines.xhtml

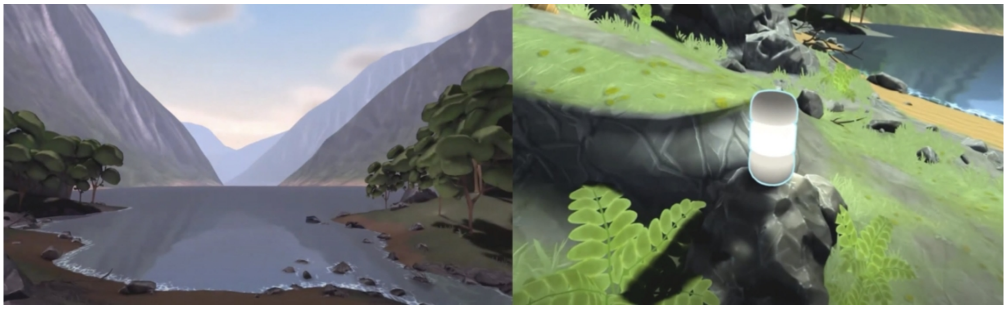

299x90mm (300 x 300 DPI)

Interview guide VR calm room

This is a semi-structured interview guide to answer this study aim (Translated from Swedish to English).

How did you experience using VR calm room?
What kind of pre-expectations did you have?
How did you experience handling the device/options in the calm room?
What kind of difference do you think using the VR calm room has had on your situation during your admission at the department?
Did you learn anything in particular from using the VR calm room, for example in the form of relaxation strategies? If so, who/which one? How?
Can you describe the process for the use of the VR calm room?
Was there anything that worried you before trying the VR calm room?
Can you describe what the ideal option for anxiety reduction would look like for you?
What can be the best help for you?
How do you think such an alternative as VR calm room can be developed?
Are there situations where you think it should not be used, if so when?
Should it be offered in other departments in healthcare?

For peer review only

COREQ (COnsolidated criteria for REporting Qualitative research) Checklist

A checklist of items that should be included in reports of qualitative research. You must report the page number in your manuscript where you consider each of the items listed in this checklist. If you have not included this information, either revise your manuscript accordingly before submitting or note N/A.

Topic	Item No.	Guide Questions/Description	Reported on Page No.
**Domain 1: Research team and reflexivity**			
Personal characteristics			
Interviewer/facilitator	1	Which author/s conducted the interview or focus group?	8
Credentials	2	What were the researcher's credentials? E.g. PhD, MD	1
Occupation	3	What was their occupation at the time of the study?	1
Gender	4	Was the researcher male or female?	N/A
Experience and training	5	What experience or training did the researcher have?	N/A
Relationship with participants			
Relationship established	6	Was a relationship established prior to study commencement?	N/A
Participant knowledge of the interviewer	7	What did the participants know about the researcher? e.g. personal goals, reasons for doing the research	8
Interviewer characteristics	8	What characteristics were reported about the inter viewer/facilitator? e.g. Bias, assumptions, reasons and interests in the research topic	8
**Domain 2: Study design**			
Theoretical framework			
Methodological orientation and Theory	9	What methodological orientation was stated to underpin the study? e.g. grounded theory, discourse analysis, ethnography, phenomenology, content analysis	7
Participant selection			
Sampling	10	How were participants selected? e.g. purposive, convenience, consecutive, snowball	7
Method of approach	11	How were participants approached? e.g. face-to-face, telephone, mail, email	7-8
Sample size	12	How many participants were in the study?	7
Non-participation	13	How many people refused to participate or dropped out? Reasons?	7
Setting			
Setting of data collection	14	Where was the data collected? e.g. home, clinic, workplace	7
Presence of non-participants	15	Was anyone else present besides the participants and researchers?	8
Description of sample	16	What are the important characteristics of the sample? e.g. demographic data, date	10
Data collection			
Interview guide	17	Were questions, prompts, guides provided by the authors? Was it pilot tested?	8
Repeat interviews	18	Were repeat inter views carried out? If yes, how many?	N/A
Audio/visual recording	19	Did the research use audio or visual recording to collect the data?	8
Field notes	20	Were field notes made during and/or after the inter view or focus group?	N/A
Duration	21	What was the duration of the inter views or focus group?	8
Data saturation	22	Was data saturation discussed?	4,9,22
Transcripts returned	23	Were transcripts returned to participants for comment and/or	N/A

For peer review only

Topic	Item No.	Guide Questions/Description	Reported on Page No.
		correction?	
**Domain 3: analysis and findings**			
Data analysis			
Number of data coders	24	How many data coders coded the data?	9
Description of the coding tree	25	Did authors provide a description of the coding tree?	9
Derivation of themes	26	Were themes identified in advance or derived from the data?	9
Software	27	What software, if applicable, was used to manage the data?	N/A
Participant checking	28	Did participants provide feedback on the findings?	N/A
Reporting			
Quotations presented	29	Were participant quotations presented to illustrate the themes/findings? Was each quotation identified? e.g. participant number	12-19
Data and findings consistent	30	Was there consistency between the data presented and the findings?	12-19
Clarity of major themes	31	Were major themes clearly presented in the findings?	11
Clarity of minor themes	32	Is there a description of diverse cases or discussion of minor themes?	11

Developed from: Tong A, Sainsbury P, Craig J. Consolidated criteria for reporting qualitative research (COREQ): a 32-item checklist for interviews and focus groups. *International Journal for Quality in Health Care*. 2007. Volume 19, Number 6: pp. 349 – 357

Once you have completed this checklist, please save a copy and upload it as part of your submission. DO NOT include this checklist as part of the main manuscript document. It must be uploaded as a separate file.

For peer review only

BMJ Open

Patient experience of a virtual reality calm room in a psychiatric inpatient care setting in Sweden: a qualitative study with inpatients

Journal:	*BMJ Open*
Manuscript ID	bmjopen-2023-076285.R2
Article Type:	Original research
Date Submitted by the Author:	27-Nov-2023
Complete List of Authors:	Ilioudi, Maria; University of Gothenburg Sahlgrenska Academy, Institute of Health and Care Sciences; Sahlgrenska universitetssjukhuset, Psychiatric Department<br>Wallström, Sara; Institute of Health and Care Sciences, Sahlgrenska Academy, University of Gothenburg; University of Gothenburg Centre for Person-Centred Care, Sahlgrenska Academy, University of Gothenburg<br>Steingrimsson, Steinn; Sahlgrenska universitetssjukhuset, Psychiatric Department; University of Gothenburg Institute of Neuroscience and Physiology, University of Gothenburg<br>Lindner, Philip; Karolinska Institutet, Centre for Psychiatry Research, Department of Clinical Neuroscience<br>Thunström, Almira Osmanovic; University of Gothenburg Institute of Neuroscience and Physiology<br>Ali, Lilas; Health and Care Sciences, Sahlgrenska academy
**Primary Subject Heading**:	Mental health
Secondary Subject Heading:	Qualitative research
Keywords:	Virtual Reality, PSYCHIATRY, MENTAL HEALTH

SCHOLARONE™
Manuscripts

I, the Submitting Author has the right to grant and does grant on behalf of all authors of the Work (as defined in the below author licence), an exclusive licence and/or a non-exclusive licence for contributions from authors who are: i) UK Crown employees; ii) where BMJ has agreed a CC-BY licence shall apply, and/or iii) in accordance with the terms applicable for US Federal Government officers or employees acting as part of their official duties; on a worldwide, perpetual, irrevocable, royalty-free basis to BMJ Publishing Group Ltd ("BMJ") its licensees and where the relevant Journal is co-owned by BMJ to the co-owners of the Journal, to publish the Work in this journal and any other BMJ products and to exploit all rights, as set out in our licence.

The Submitting Author accepts and understands that any supply made under these terms is made by BMJ to the Submitting Author unless you are acting as an employee on behalf of your employer or a postgraduate student of an affiliated institution which is paying any applicable article publishing charge ("APC") for Open Access articles. Where the Submitting Author wishes to make the Work available on an Open Access basis (and intends to pay the relevant APC), the terms of reuse of such Open Access shall be governed by a Creative Commons licence – details of these licences and which Creative Commons licence will apply to this Work are set out in our licence referred to above.

Other than as permitted in any relevant BMJ Author's Self Archiving Policies, I confirm this Work has not been accepted for publication elsewhere, is not being considered for publication elsewhere and does not duplicate material already published. I confirm all authors consent to publication of this Work and authorise the granting of this licence.

For peer review only

Patient experience of a virtual reality calm room in a psychiatric inpatient care setting in Sweden: a qualitative study with inpatients

Authors: Maria Ilioudi[1,2], Sara Wallström[1,3,4,5], Steinn Steingrimsson[1,6], Philip Lindner[7], Almira Osmanovic Thunström[1,6], Lilas Ali[1,2,3]

Author affiliations

[1] Region Västra Götaland, Sahlgrenska University Hospital, Psychiatric Department, Gothenburg, Sweden

[2]Institute of Health and Care Sciences, Sahlgrenska Academy, University of Gothenburg, Gothenburg, Sweden

[3]Centre for Person-Centred Care (GPCC), Sahlgrenska Academy, University of Gothenburg, Gothenburg, Sweden

[4]Region Västra Götaland, Sahlgrenska University Hospital, Department of Forensic Psychiatry, Gothenburg, Sweden

[5]Centre for Ethics, Law and Mental Health (CELAM), University of Gothenburg, Gothenburg, Sweden

[6]Institute of Neuroscience and Physiology, University of Gothenburg, Sahlgrenska Academy, Gothenburg, Sweden

[7]Centre for Psychiatry Research, Department of Clinical Neuroscience, Karolinska Institute and Stockholm Health Care Services, Stockholm, Sweden

ORCID iDs

Maria Ilioudi, maria.ilioudi@gu.se

Orchid: 0000-0003-0709-7768

For peer review only

Sara Wallström, sara.wallstrom@gu.se

Orchid: 0000-0001-7579-4974

Steinn Steingrimsson, steinn.steingrimsson@neuro.gu.se

Orchid: 0000-0001-8320-5607,

Philip Lindner, philip.lindner@ki.se

Orchid: 0000-0002-3061-501X

Almira Osmanovic Thunström, almira.osmanovic.thunstrom@gu.se

Orchid: 0000-0002-8791-9524

Lilas Ali, lilas.ali@gu.se

Orchid: 0000-0001-7027-4371

Correspondence to: Assoc. Prof. Steinn Steingrimsson, MD, PhD, Department of Psychiatry, Sahlgrenska University Hospital, Region Västra Götaland, 41650 Gothenburg, Sweden; Tel: 46 313421000: Email: steinn.steingrimsson@neuro.gu.se

Manuscript type: Original research

Abstract length: 287

Text length: 5378

No. of references: 50

No. of graphics: 3 (2 tables/1 figure)

Keywords: mental health, psychiatry, virtual reality, qualitative design

For peer review only

ABSTRACT

Objective: Calm rooms have been developed and implemented in psychiatric inpatient care settings to offer patients a dedicated space for relaxation in a convenient and safe environment. Recent technology developments have enabled virtual reality (VR) equivalents of calm rooms that can be feasibly deployed in psychiatric care settings. While research has shown VR environments to be efficacious in inducing relaxation, little is known how these virtual calm rooms are perceived by patients. The aim of this study was to elucidate patient experiences of using a VR calm room in a psychiatric inpatient setting.

Design: Qualitative interview study. Semi-structured interviews were analysed using qualitive inductive content analysis, which focuses on the interpretation of texts for making replicable and valid inferences.

Setting: Swedish hospital psychiatric inpatient care setting with a wireless, three degrees-of-freedom VR head-mounted display running a calm room application simulating nature environment.

Participants: 20 adult patients (12 women) with bipolar disorder (n=18) or unipolar depression (n=2).

Results: Participants experienced the use of the VR calm room as having a positive impact on them, inducing awareness, calmness and well-being. They were thankful to be offered a non-pharmacological alternative for anxiety relief. Participants also expressed that they had some concerns about how they would react emotionally before using the VR device. However, after use, they highlighted that their overall experience was positive. They also expressed that they could see potential for further development of VR technology in psychiatric care.

For peer review only

Conclusions: VR technology has the potential to solve pressing logistic issues in offering calm rooms in psychiatric inpatient care. VR calm rooms appear to be appreciated by psychiatric inpatients, who value their accessibility, convenience and variety of modalities offered. Participants perceived an increase in their well-being after use.

Keywords: virtual reality, psychiatry, relaxation, psychiatric inpatient care, qualitative design, interviews

For peer review only

Strengths and limitations of this study

- This study was conducted in an inpatient psychiatric setting with participants who were introduced to a VR calm room as a new method for relaxation.
- The interview data in this study provided varied descriptions of patient experiences of using the VR calm room.
- There were some difficulties in engaging patients in longer interviews, which was not unexpected given the acute phase that patients were in at that time.
- The short duration of engagement by the informants during interviews resulted in more inclusions (n=20) to have enough data to draw necessary conclusions.
- VR calm room has the potential to be a cost-effective, non-pharmacological form of anxiety relief in inpatient psychiatric setting.

For peer review only

INTRODUCTION

Patients admitted to psychiatric inpatient wards often experience episodes of heightened emotions, which left uncared for may ultimately necessitate invasive methods such as forced medication, restraints, and one-on-one monitoring, in order to keep them from harming themselves. So called calm rooms (sensory rooms) offer a seemingly attractive alternative to these invasive methods, and consist of multisensory environments that stimulate sensation, olfactory, auditory, vision and, can be decorated with elements such as lights, pictures, iPad, aromatic oils, blankets.[1] The purpose is to offer patients a dedicated space for relaxation, in order to induce well-being through calming exercises and distraction from distress. Guided relaxation and other instructed exercises may also be offered.[2,3,4,5] Calm rooms can also be used to provide self-help through a convenient and safe environment and, at the same time, empower the therapeutic relationship between the patient and healthcare workers at inpatient units.[1,6] Previous research indicates that calm rooms can be used for clinical purposes as a beneficial method for patients in reducing stress and inducing well-being.[1, 4, 5, 7, 8]

Despite well-known benefits, calm rooms are far from ubiquitous in psychiatric inpatient wards, presumably due to the logistic required: there needs to be a dedicated room (with an overall low occupation rate), fitted with special décor and equipment, and special usage procedures are required. Recently, research has begun exploring whether Virtual Reality (VR) technology can be used to create virtual calm rooms. VR refers computer-generated, perceived three-dimensional and gaze-interactive environments, typically through the use of a head-mounted display.[9] VR technology is increasingly being used in the treatment of mental disorders and numerous interventions have been developed and

For peer review only

tested.[10,11,12,13,14] The term "virtual reality" has been used to describe a VR environments may promote relaxation through visualisation, engagement and immersion into pleasant virtual environments (often nature simulations), and by removing users from stressful situations.[15]

There are some clinical studies involving VR calm rooms in psychiatric settings, which show the efficacy of VR environments for mental health conditions. [8,16,17,18] Moreover, studies have also shown the efficacy [19,20,21] of VR in treating mental conditions, including social anxiety disorder,[22,23] post-traumatic stress disorder,[24] generalised anxiety disorder (GAD) [8] and phobias.[12,25] VR technology has also been used to provide immersive experiences for relaxation.[26,27,28,29] A previous study has suggested that VR nature relaxation immediately reduced negative affective states and psychological stress in patients with psychiatric disorders.[18] In addition, Repetto et. al.[8] showed, in a VR pilot study for the treatment of GAD, that using VR may result in better clinical outcomes at the end of the treatment. The participants also answered that they were very satisfied to use the device in every-day life's situations to relax.[8]

VR technology solves logistic issues since it can be used anywhere [17, 20] and is affordable in comparison to refurbishing a room at a psychiatric inpatient care setting.[12] The technology has also been shown to be convenient and safe in psychiatric inpatient care settings since it is made of harmless materials and is usually used with supervision until the patient is trusted on his or her own.[30] VR relaxation settings provide a sense of presence, pleasure, activation and engagement, and a personalised experience.[31] However, the appropriateness of VR interventions in acute conditions has not been studied extensively. In addition, the healthcare professionals need training and involving the multidisciplinary team in

For peer review only

decision-making for VR implementation in the ward. Both patients and staff expressed a practical issue about the access to private space to provide the VR application [32]

There is a lack of knowledge of how users in psychiatric inpatients perceive the use of VR-based relaxation technologies. Therefore, the aim of this study was to elucidate patient experience of using a VR calm room in psychiatric inpatient care.

MATERIAL AND METHODS

Study design

This study had a qualitative and descriptive design. Semi-structured interviews were conducted and analysed using a qualitive content analysis based on inductive theory. This study was part of a larger research project aimed at evaluating the effect of VR and physical calm rooms in psychiatric inpatient care.[2]

Setting and participants

Inclusion criteria were age ≥18 years, admitted for psychiatric inpatient care at a care setting specialised in bipolar disorder in the western region of Sweden (Sahlgrenska University Hospital, Gothenburg) and having previously used the VR calm room at least one time. Due to a lack of empty beds in other wards, patients with other psychiatric diagnoses were also included in this study. A total of 25 patients fulfilled the inclusion criteria and were asked to participate in the study by the first author. Of those, 5 declined and 20 agreed to an interview. There was no overlap in samples between the current study and a previously reported trial.[2]

For peer review only

Procedure

The VR calm room was available at all times on the ward for the patients to use after consulting with the staff. After the first use the patients were asked to participate in the study. Patients who used the VR calm room received instructions on the VR headset's functions and how to navigate in the virtual environment. To engage with the device, patients used a wireless, three degrees-of-freedom VR head-mounted display (an Oculus Go) running the calm room application developed by Mimerse. Example screenshots of the environment are shown in figure 1. Patient representatives were involved in the design process of the VR environment. The application offers a selection of different soothing nature environments (e.g. a desert, a forest beach) that can be tailored through, for example, the day and night cycle and dynamic weather. In addition, users may engage in breathing exercises, mindfulness programs and relaxing music. The user can interact with the environment by looking at different objects inside the program and using the handheld controller to adjust preferences and build individualised scenery. The interactive smorgasbord approach was chosen in light of research showing that users have different preferences for different virtual nature environments [33], as well as non-VR research showing the importance of matching soothing music to user preferences.[34]

Patient and public involvement

No patient involved

For peer review only

Data collection

The individual interviews all followed a semi-structured interview-guide developed by the authors. The interviews were conducted between October 2021 and May 2022 by the first author (MI). Interviews took place at the psychiatric inpatient care setting during patients' care time. The mean duration of the interviews was 15.15 (range, 10–24) minutes. The interviews were recorded and transcribed verbatim. The participants were asked, for example, how they experienced the calm room, was there anything that worried them when using the VR calm room, how do they think an alternative to the VR calm room can be developed and what kind of difference do they think using the VR calm room has made for their situation. After 20 interviews, data saturation was attained.

Data analysis

Data was analysed using qualitative inductive content analysis, which search for patterns in the text, aiming to acquire a theoretical understanding from collected interview data.[35] The inductive approach entails that text is analysed based on the participants' narratives through grouping meaning units containing aspects related to each other in content and context.[36] Baxter [37] defines 'meaning unit' as the constellation of words or statements that relate to the same central meaning. A condensed description of the phenomenon is achieved by emphasising differences and similarities within the data. A theme is a thread of an underlying meaning through meaning units or codes on an interpretive level. Meaning-units were identified according to the study aim, which were then condensed and labelled with codes. [37]

For peer review only

First, the first and last authors (MI and LA) familiarised themselves with the material by reading the transcriptions several times in order to grasp the central meaning of the texts. Through careful reading, relevant sentences and phrases to the study aim were identified. These were then used in the constellation of meaning units and to begin the coding process. The codes were then sorted and abstracted into themes and subthemes. To ensure trustworthiness of the themes, the first and last author worked closely with the analysis. The other authors validated the text and discussed the themes and subthemes until consensus was reached.

Ethical considerations

Ethical approval was granted by the Swedish Ethical Review Authority (Dnr: 318-18). All participants received verbal and written information about the study and that they could end the interview at any time without specifying a reason. All data were treated confidentially. All ethical decisions were guided by the Declaration of Helsinki [38] and its subsequent amendments The participants admitted to the psychiatric inpatient care setting may have been under compulsory psychiatry care. The authors were aware that the participants' mental health condition might be affected and were prepared to interrupt the research process or offer suitable support services upon participant request or if this was deemed necessary.

RESULTS

A total of 20 patients (12 women and 8 men) participated in the study. Participant characteristics are summarised in table 1.

Table 1 Patient demographic and clinical characteristics (n=20)

Variable	No. of patients
Sex	

For peer review only

Men	8
Women	12
Age, years	
20–29	4
30–49	5
50–59	5
60–69	6
Diagnosis	
Bipolar disorder	18
Unipolar depression	2

The first overall impression of the analysis revealed that using the VR calm room had a positive impact on the participants during their period of care at the inpatient psychiatric unit. The experience raised feelings of awareness, calmness and well-being. Participants also had suggestions on further development of the VR technology in mental healthcare in the future. An overview of the themes and sub-themes is presented in table 2.

Table 2 Themes and sub-themes from interviews following VR calm room use

Feelings before and during use	Negative point of view	Potential for future development
Being offered an alternative to medication	Practical issues and side effects	Willing to continue use
Feelings of relief and relaxation	Unpleasant experiences	Recommending VR
Sense of independency		

For peer review only

Feelings before and during use

Participants expressed that they had a positive attitude about using the calm room before the intervention and thought it would be an enjoyable part in the overall time of care at the psychiatric inpatient unit. Several participants mentioned that they preferred non-pharmacological alternatives such as VR. Furthermore, they experienced a sense of independence and gave positive feedback about the opportunity to learn new relaxation methods.

Being offered an alternative to medication

During inpatient psychiatric care, the participants sometimes encountered boundaries in being able to achieve a peaceful state of mind on their own due to the care environment being hectic or perceived as stressful. They perceived there to be few non-pharmacological alternatives available, or any support offered to them to enhance their well-being and reduce anxiety. They therefore expressed gratitude over being offered the use the VR device as an alternative to drugs.

The participants also mentioned that use of the virtual calm room was very anxiety-relieving and therefore had a positive impact on their well-being during the time they were admitted to the inpatient psychiatry unit. Being admitted to psychiatric inpatient care was typically associated with high levels of anxiety and feelings of seclusion. The participants highlighted that inpatient psychiatric care needs alternative methods that they can be offered for anxiety reduction and self-support. The participants also stated that they were upset about the limited options as well as the traditional relaxation methods previously available and therefore really appreciated the time that they spent in the VR calm room. The VR calm room enriched their day at the psychiatric inpatient care setting by giving them a new

For peer review only

dimension to rely on when needed. The participants described the VR rooms as a strategy with which they could find respite and develop emotional self-care in a stressful environment at the psychiatric inpatient care setting. Participants also highlighted that the VR represented something innovative in psychiatric care which gives hope for new treatment options.

> "…one thing is that we have a lot of time here, it's slow, you have nothing to do, you maybe feel bad, if it's anxiety. That you get a tool and you sit for a certain time. I think that can help, because sometimes you might just go and go and go and do nothing, then it doesn't get better, there are no…you have to have multiple tools. This…virtual room, well, I think it's a good thing..." P1

"…But I had allot of conversations before, frequently, but it wasn't enough. But it is this with less stress and conversations. It is what I mostly believe in. But…and now this, it is a fantastic point of direction, it's what it is. Really…" P3

Feelings of relief and relaxation

Participants had varying experiences of how VR could potentially influence their mental condition in a better way. The use was described as being helpful for their condition and that it gave them a sense of a holistic approach to their feelings. They expressed feelings of joy and that it was something beautiful to experience. Participants also stated that using the VR calm room fulfilled their day because they felt that they had done something creative and worthwhile for their condition. They described the environment they experienced in the VR device as dynamic and that they found some kind of a new dimension to strategically enhance their well-being. Participants also experienced the VR environment to induce a state of calm and

For peer review only

creating an awareness of their emotions at the time of use. They found it comfortable to go into another reality and leave the physical one behind for a while. Participants in this study usually experienced a stressful reality on a daily basis which contributed to their feelings of anxiety.

"...it felt very nice to enter another world and then that I felt calmer afterwards…"

P1

"…I thought I was on an island, anyways, and like, I enjoyed that there were mountain peaks, it was nature, it was leaves and it was sand and sea, or water. And it was light and it was dark and it was like morning, day dusk, night, sky. Yes, everything one needs…"

P13

Sense of independency

The VR device was viewed as an opportunity for participants to learn new relaxation methods, such as relaxing the body, practicing breathing and maintaining positive thinking. Participants expressed that it was easy to learn and practice guided meditation using the VR application. Furthermore, they mentioned that VR was easier to handle compared to usual relaxation methods that they would use in the "real world". Participants wished to stay longer in the VR calm room because they described their use as a new way to reach a feeling of calmness. Participants also expressed that using the VR room gave them a sense of independence in the psychiatric inpatient care setting by allowing them to make their own decisions and becoming more involved in their own care.

For peer review only

"…It's not easy to describe. Now I'll see, you first came...completely shocked by that nature and then feelings just grow, it's nice inside you that fills up the soul…" P7

Participants indicated that the device was comfortable and flexible to use. They could, on their own, decide how they wanted their calm room to be and the duration of the session. This was all handled by themselves, which gave them a sense of independence in the psychiatric inpatient unit. Furthermore, participants expressed that being able to stay in the room at the psychiatric inpatient care setting solved logistic issues and could therefore be used at any place in the ward based on their own individual preferences. They also appreciated that they could pause, end and restart the session at any time.

"…If you're alone, if no staff is here, the VR glasses would have been good because then you can also lie down. I thought that was good. In this relaxation thing, they don't nag. In some relaxation videos it's, tie the hand, open the hand, tie, open. Here it was just, tie the hand and then you did the rest yourself. So that was enough information…" P5

Negative point of view

This theme describes the participants' negative experiences and feelings related to the use of the VR device. Some experienced unpleasant feelings at the start when first using the VR calm room. They were afraid and worried about some components of the VR nature environment such as the darkness and birds. Some of the

For peer review only

participants also mentioned that the device was not very comfortable to wear on their head.

Practical issues and side effects

Participants experienced negative issues with the VR device being uncomfortable and heavy to carry for a longer period of time on their head. Some participants mentioned that the VR device did not fit properly and would therefore slip during the time they used it. This affected the overall experience of VR device use. They also mentioned that sometimes they would become dizzy and feel nauseous while using the device. Participants also mentioned that these issues usually came at the start of the session and would become less common as they became more familiar with the VR environment. None of the participants interrupted their session due to cybersickness. Before using the VR device, they had their hesitations, some of them worried that they might feel a bit dizzy and uncomfortable. However, they managed to overcome that, after using the VR device for a while, finding it was not as frightening or strange as they thought that it would be. The participants expressed that, when they used the VR device, they felt more relaxed at the end of the session than before.

> "...but that it slipped and then it got on the glasses and then there was no sharpness so I had to take it up all the time..." P3

"… In the beginning it was difficult to relax, I thought, in the beginning I kind of was laying and kind of tossed and turned like this … and then when some time had passed I became really relaxed and then the rain came and birds and it was… I actually forgot where I was…"P17

For peer review only

Unpleasant experiences

Entering the VR world was for some participants an unpleasant experience because they were sometimes stressed and worried for no reason that they can recall. They were also expecting their own imagination to kick in and that is why they did not want to be left alone in the VR calm room. They indicated that they were waiting for more life and interaction in the virtual world such as with animals or other creatures.

"…At first it was a bit strange and scary because when I turned my head there was like nothing but the VR environment and then I got stressed at first because I felt it was too close and I didn't recognise myself. But then it got better and then it became a bit like my environment, my own. So it turned around and got better..." P5

Furthermore, the analysis indicated that some of the participants experienced VR as frightening and an unsafe environment when specific images would appear such as birds flying everywhere or something else unexpected that might happen in the VR environment making them stay alert in case something unexpected would occur. Participants also described that one scenario in the VR was too dark, which usually led to them becoming anxious of the light and therefore interpreting the experience as being uncomfortable as it would remind them of walking into death when you see the light at the end of the session.

"...I perceived the light as...a bit like death, that you walk towards the light, that it became too much...that it became too peaceful..." P4

For peer review only

"…Then the birds, there were so many birds, it didn't feel like a threat but it felt a bit stressful, I don't like seagulls, so it got a bit like this..." P15

Potential for future development

This theme captures participants' suggestions and recommendation on future use of a VR calm room and what kind of potentials they see for further development, for example, VR as an alternative to treatment for stress reduction.

Willing to continue use

The VR experience was described as a new experience for the participants. They stated that they would consider using the VR device again, both in a healthcare setting and by themselves for self-care at home. They wanted to have easy accessibility to the VR device every day in the psychiatric inpatient care setting and wished that there would be several VR devices available for use. Furthermore, the participants expressed that they wished to use VR on a daily basis as a part of their treatment. Several participants wanted to have a VR device of their own and asked if they could buy one or get access to the calm room application. The main statement that was given by the participants in this study was that they wanted to use the device again in the future.

”…This was really relaxing.. I wish that it was always available to us that are bipolar or has other mental problems, to be used for treatment purpose…” P19

"…I want to use it again and I was still sceptical before, I was open-minded, sceptical, but I would like to use it again if I get the chance..." P20

For peer review only

Recommending VR

Participants mentioned that VR could reduce anxiety and induce well-being. They recommended the use of the VR device for therapeutic purposes as they could see potential for that. Despite some frightening features, the participants also described use as a positive and not harmful experience, which is why they would recommend it to others.

"…It's relaxing so it affects everyone positively, it can be positive for everyone..." P7

However, there were some participants who mentioned that they thought that the VR concept might be inappropriate for patients who have vision problems and difficulties with crisis situations, such as for some patients with psychosis. It might also be inappropriate to use when feeling too tired or trapped. Participants wished for further functions to be developed in the VR device and wanted to be able to choose different environments, not just the natural calm room that was programmed. They wanted to experience more dynamics, and visions that included more animals and other creatures than what they had experienced. The participants recommended and wished to use the VR for about 15 to 20 minutes as they felt that in that time, they could experience the full effect of anxiety relief.

DISCUSSION

The aim of this study was to elucidate experiences of using a VR calm room by patients admitted to psychiatric inpatient care. Our findings strengthen the results of

For peer review only

previous research, which showed VR calm rooms to increase well-being and lower distress. [26,28,39] In the current study, participants expressed feelings of relaxation, which induced their well-being, when using the VR room. Implementation of VR technology in psychiatric inpatient care settings has been shown to successfully improve patient recovery and emotional self-care [40] as a VR calm room strengthens patient choices, leading to a flexible and convenient strategy for resolving distress in an acute psychiatric setting. [2] Such methods might enhance patient engagement in their care and to take on the responsibility for recovery of their severe mental condition.

The findings support that the VR calm room reduces the experience of stress and helped patients to feel better with a sense of calm. Previous studies have shown that immersion into VR environments has positive psychological and physical effects.[17, 41] In addition, VR calm room design can distract patients from stressful acute psychiatric settings.[42] The design of the VR room in our study, which aimed at creating a less hospital-like experience with features supporting familiarity and flexibility, may have contributed to the patients' sense of safety, self-control and enjoyment. Exposure to VR nature images can provide emotional well-being for patients who do not have access to outdoor activities and nature.[43] Patients stay in psychiatric inpatient care for long periods of time and VR calm rooms have the potential to offer visual access to nature and its positive effects. VR technology may solve logistics issues in the psychiatric inpatient care as patients can use the VR device in their own room in the psychiatric inpatient setting without affecting safety.[10,44] In comparison to traditional relaxation methods, participants mentioned that the VR calm room was easy to access and use. VR relaxation is

For peer review only

appreciated by users,[45] and has become affordable and more accessible, which has led to its increasingly widespread use in psychiatric care.[42]

Participants reported several negative experiences with the use of VR. Some patients expressed that the experience was unpleasant and them being worried for no reason. They also mentioned becoming dizzy and that some of the images in the VR calm room were scary. The unpleasant experiences might be due to the new environment and unfamiliar components. Our findings align with previous studies where users reported issues with the installation process, overheating, the visual presentation and negative effects such as anxiety.[10] It is possible that some of these negative experiences may be due to personal preferences, which might be avoidable by offering the user a variety of environments.[46] Patients admitted to psychiatric inpatient care experience low satisfaction with their influence on the choice of treatment and medication.[47] Non-pharmacological methods such as physical calm rooms have safety issues; therefore, healthcare workers may become risk averse and initially worried about letting patients stay on their own in calm rooms. [48] However, activities that break up the monotony in psychiatric inpatient care settings were appreciated by the patients.[49]

The participants in this study highlighted some future recommendations and suggestions about VR calm rooms such as creation of several scenarios and device options with more modalities. This is in line with previous research which suggests that the VR scenario should be expanded to include various additional experiences such as live music scenes, scenes featuring people walking around and animals.[50] Patients recommended the use of the VR device as a therapeutic method for relaxation and stress reduction both in healthcare settings and for patients to use on their own at home. However, some participants mentioned that VR might be

For peer review only

inappropriate for patients with vision problems and when being too tired. Also, some recommended avoiding the use of VR in crisis psychiatric conditions due to the high levels of anxiety and lower adaptation ability in these circumstances. Self-relaxation is an important feature of the care process in psychiatric inpatient care; therefore, participants stated that they wanted to use the VR room in the future as a relaxation tool. Broader use of VR calm rooms should be investigated to explore their potential for being part of usual treatment in acute psychiatric care with individualized content. Important considerations in inpatient setting are agitation and risk for violence, which have to be evaluated in each case. Presumably, a patient that is used to the technique might benefit in an agitated state but studies on the safety of such an intervention are needed.

Study strengths and limitations

The primary strength of this study is that it was conducted in inpatient psychiatric care. It is especially important to include patients who are diagnosed with severe acute psychiatric conditions as they are often excluded from studies due to their illness. This study had a sample of participants with bipolar diagnosis and unipolar depression. The interview data in this study provided varied descriptions of patient experiences of using the VR calm room.

The qualitative approach using content analysis [35,36,37] made it possible to elucidate patient experiences using a VR calm room in acute psychiatric care. Trustworthiness was established through including all authors' reflection and engagement in all steps of the analyses. Regarding dependability, the same author conducted the interviews using a semi-structured interview guide. One limitation in this study was the difficulties to engage patients in the interviews for a longer period

For peer review only

of time. The interview time ranged between 10 and 24 minutes (mean 15.15). This might be due to the acute phase that patients were in at the time of their interviews and the severity in their illness; however, this was compensated by the inclusion of more participants in the study to have enough data to draw necessary conclusions. Patient representatives were involved in the design process of the VR environment, but there was no patient involvement in the subsequent processes of the study.

Conclusion

A VR calm room provides a cost-effective, modern, convenient method for relaxation in acute psychiatric care. Patients appreciated the accessibility, convenience and variety of modalities. They also thought that it increased their well-being. VR technology provides potentialities in solving logistic issues and is cost effective. Further research is needed that includes patients in psychiatric inpatient care with a broader range of mental conditions.

For peer review only

Acknowledgements We thank the participants who generously shared their experiences in this study. The authors would also like to thank the Psychiatry Affective Department, the managers and, particularly, area manager Mathias Alvidius for their support and assistance in conducting the study. We would also like to thank William Hamilton who developed the virtual reality calm room scenario. We would also like to thank Peter Todd (Tajut Ltd., Kaiapoi, New Zealand) for third-party editorial assistance in drafting of this manuscript, for which he received financial compensation.

Contributors SS, PL, AT and LA were involved in the conceptualization of the study. LA, SW and MI developed the methodology. MI curated the study data. MI, LA and SW conducted the formal analysis. MI, LA and SW undertook administration of the project. MI and LA wrote the first draft of the article. All authors were involved in the conduct of the study, review and editing of the article, and approved the submitted version.

Funding The project was funded by Lokala FoU-rådet i Göteborg och Södra Bohuslän with 110,000 Swedish krona.SS received funding from The Söderström König Foundation (SLS-974247).

Competing interests PL reports having received minor consulting fees from Mimerse, the company that developed the VR application used. Mimerse is no longer an active company. The other authors declare that they have no competing interests.

For peer review only

Patient consent for publication Not applicable.

Ethics approval Ethical approval was granted by the Swedish Ethical Review Authority (Dnr: 318-18). Written consent was obtained from each participant.

Provenance and peer review Not commissioned, externally peer reviewed.

Data availability statement Due to local regulations, we are not able to share the data freely but raw data can be provided from the corresponding author on reasonable request.

For peer review only

REFERENCES

1 Björkdahl A, Perseius KI, Samuelsson M, *et al*. Sensory rooms in psychiatric inpatient care: staff experiences. *Int J Ment Health Nurs* 2016;25:472–9. https://doi.org/10.1111/inm.12205

2 Ilioudi M, Lindner P, Ali L, et al. Physical Versus Virtual Reality–Based Calm Rooms for Psychiatric Inpatients: Quasi-Randomized Trial. *Journal of Medical Internet Research 2023; 25*. https://doi.org/10.2196/42365

3 Andersen C, Kolmos A, Andersen K, *et al*. Applying sensory modulation to mental health inpatient care to reduce seclusion and restraint: a case control study. *Nord J Psychiatry* 2017;71:525–8. https://doi.org/10.1080/08039488.2017.1346142

4 Cummings KS, Grandfield SA, Coldwell CM. Caring with comfort rooms. Reducing seclusion and restraint use in psychiatric facilities. *J Psychosoc Nurs Ment Health Serv* 2010;48:26–30. https://doi.org/10.3928/02793695-20100303-02

5 Novak T, Scanlan J, McCaul D, *et al*. Pilot study of a sensory room in an acute inpatient psychiatric unit. *Australas Psychiatry* 2012;20:401–6. https://doi.org/10.1177/1039856212459585

6 Seckman A, Paun O, Heipp B, *et al*. Evaluation of the use of a sensory room on an adolescent inpatient unit and its impact on restraint and seclusion prevention. *J Child Adolesc Psychiatr Nurs* 2017;30:90–7. https://doi.org/10.1111/jcap.12174

For peer review only

7 Lindner P, Miloff A, Bergman C, *et al*. Gamified, automated virtual reality exposure therapy for fear of spiders: a single-subject trial under simulated real-world conditions. *Front Psychiatry* 2020;11:116. https://doi.org/10.3389/fpsyt.2020.00116

8 Repetto C, Gaggioli A, Pallavicini F, *et al*. (2011). Virtual reality and mobile phones in the treatment of generalized anxiety disorders: a phase-2 clinical trial. *Personal and Ubiquitous Computing* 2011;17:253–60. https://doi.org/10.1007/s00779-011-0467-0

9 Scarfe P, Glennerster A. The science behind virtual reality displays. *Annu Rev Vis Sci* 2019;5:529–47. https://doi.org/10.1146/annurev-vision-091718-014942

10 Fagernäs S, Hamilton W, Espinoza N, *et al*. What do users think about Virtual Reality relaxation applications? A mixed methods study of online user reviews using natural language processing. *Internet Interv* 2021;24:100370. https://doi.org/10.1016/j.invent.2021.100370

11 Lloyd A, Haraldsdottir E. Virtual reality in hospice: improved patient well-being. *BMJ Support Palliat Care* 2021;11:344–50. https://doi.org/10.1136/bmjspcare-2021-003173

12 Miloff A, Lindner P, Hamilton W, *et al*. Single-session gamified virtual reality exposure therapy for spider phobia vs. traditional exposure therapy: study protocol for a randomized controlled non-inferiority trial. *Trials* 2016;17:60. https://doi.org/10.1186/s13063-016-1171-1

13 Tarrant J, Viczko J, Cope H. Virtual reality for anxiety reduction demonstrated by quantitative EEG: a pilot study. *Front Psychol* 2018;9:1280. https://doi.org/10.3389/fpsyg.2018.01280

For peer review only

14 Thunström AO, Sarajlic Vukovic I, Ali L, *et al*. Prevalence of virtual reality (VR) games found through mental health categories on STEAM: a first look at VR on commercial platforms as tools for therapy. *Nord J Psychiatry* 2022;76:474–85. https://doi.org/10.1080/08039488.2021.2003859

15 Riches S, Azevedo L, Bird L, *et al*. Virtual reality relaxation for the general population: a systematic review. *Soc Psychiatry Psychiatr Epidemiol*

16 Freeman D, Bradley J, Antley A, *et al*. Virtual reality in the treatment of persecutory delusions: randomised controlled experimental study testing how to reduce delusional conviction. *Br J Psychiatry* 2016;209:62–7. https://doi.org/10.1192/bjp.bp.115.176438

17 Lindner P, Dagöö J, Hamilton W, *et al*. Virtual Reality exposure therapy for public speaking anxiety in routine care: a single-subject effectiveness trial. *Cogn Behav Ther* 2021;50:67–87. https://doi.org/10.1080/16506073.2020.1795240

18 Veling W, Lestestuiver B, Jongma M, *et al*. Virtual reality relaxation for patients with a psychiatric disorder: crossover randomized controlled trial. *J Med Internet Res* 2021;23:e17233. https://doi.org/10.2196/17233

19 Carl E, Stein AT, Levihn-Coon A, *et al*. Virtual reality exposure therapy for anxiety and related disorders: a meta-analysis of randomized controlled trials. *J Anxiety Disord* 2019;61:27–36. https://doi.org/10.1016/j.janxdis.2018.08.003

20 Fodor LA, Coteț CD, Cuijpers P, *et al*. The effectiveness of virtual reality based interventions for symptoms of anxiety and depression: a meta-analysis. *Sci Rep* 2018;8:10323. https://doi.org/10.1038/s41598-018-28113-6

21 Wechsler TF, Kümpers F, Mühlberger A. Inferiority or even superiority of virtual reality exposure therapy in phobias? A systematic review and quantitative meta-analysis on randomized controlled trials specifically comparing the efficacy of

For peer review only

virtual reality exposure to gold standard *in vivo* exposure in agoraphobia, specific phobia and social phobia. *Front Psychol* 2019;10:1758. https://doi.org/10.3389/fpsyg.2019.01758

22 Anderson PL, Price M, Edwards SM, *et al*. Virtual reality exposure therapy for social anxiety disorder: a randomized controlled trial. *J Consult Clin Psychol* 2013;81:751–60. https://doi.org/10.1037/a0033559

23 Bouchard S, Dumoulin S, Robillard G, *et al*. Virtual reality compared with *in vivo* exposure in the treatment of social anxiety disorder: a three-arm randomised controlled trial. *Br J Psychiatry* 2017;210:276–83. https://doi.org/10.1192/bjp.bp.116.184234

24 Reger GM, Koenen-Woods P, Zetocha K, *et al*. Randomized controlled trial of prolonged exposure using imaginal exposure vs. virtual reality exposure in active duty soldiers with deployment-related posttraumatic stress disorder (PTSD). *J Consult Clin Psychol* 2016;84:946–59. https://doi.org/10.1037/ccp0000134

25 Botella C, Fernández-Álvarez J, Guillén V, *et al*. Recent progress in virtual reality exposure therapy for phobias: a systematic review. *Curr Psychiatry Rep* 2017;19:42. https://doi.org/10.1007/s11920-017-0788-4

26 Annerstedt M, Jönsson P, Wallergård M, *et al*. Inducing physiological stress recovery with sounds of nature in a virtual reality forest: results from a pilot study. *Physiol Behav* 2013;118:240–50. https://doi.org/10.1016/j.physbeh.2013.05.023

27 Baños RM, Botella C, Guillen V, *et al*. An adaptive display to treat stress-related disorders: EMMA's world. *British Journal of Guidance and Counselling* 2009;37:347–56. https://doi.org/10.1080/0306988090297064

For peer review only

28 Lindner P, Miloff A, Hamilton W, *et al*. The potential of consumer-targeted virtual reality relaxation applications: descriptive usage, uptake and application performance statistics for a first-generation application. *Front Psychol* 2019;10:132. https://doi.org/10.3389/fpsyg.2019.00132

29 Serrano B, Baños RM, Botella C. Virtual reality and stimulation of touch and smell for inducing relaxation: a randomized controlled trial. *Computers in Human Behavior* 2016;55:1–8. https://doi.org/10.1016/j.chb.2015.08.007

30 Dascal J, Reid M, IsHak WW, *et al*. Virtual reality and medical inpatients: a systematic review of randomized, controlled trials. *Innov Clin Neurosci* 2017;14:14–21. PMID: 28386517

31 Pardini S, Gabrielli S, Dianti M, *et al*. The role of personalization in the user experience, preferences and engagement with virtual reality environments for relaxation. *Int J Environ Res Public Health* 2022;19:7237. https://doi.org/10.3390/ijerph19127237

32 Brown P, Waite F, Lambe S, *et. al*. Automated Virtual Reality Cognitive Therapy (gameChange) in Inpatient Psychiatric Wards: Qualitative Study of Staff and Patient Views Using an Implementation Framework. *JMIR Formative Research* 2022; *6*:34225. https://doi.org/10.2196/34225

33 Gao T, Zhang T, Zhu L, *et. al*.. Exploring psychophysiological restoration and individual preference in the different environments based on virtual reality. *International Journal of Environmental Research and Public Health* 2019; *16*:3102 https://doi.org/10.3390/ijerph16173102

34 Jiang J, Zhou L, Rickson D, *et al.* (2013). The effects of sedative and stimulative music on stress reduction depend on music preference. *The Arts in Psychotherapy*, 2013:40; 201-205. https://doi.org/10.1016/j.aip.2013.02.002

35 Krippendorff, K. Content Analysis: an Introduction to its Methodology, 4th ed. Thousand Oaks, CA: Sage Publications 2018.

36 Graneheim UH, Lundman B. Qualitative content analysis in nursing research: concepts, procedures and measures to achieve trustworthiness. *Nurse Educ Today* 2004;24:105–12. https://doi.org/10.1016/j.nedt.2003.10.001

37 Baxter LA. Content analysis. In: Montgomery BM, Duck S, eds., Studying Interpersonal Interaction. The Guilford Press, New York, NY 1991:239–54.

38 World Medical Association. World Medical Association Declaration of Helsinki: ethical principles for medical research involving human subjects. *JAMA* 2013;310:2191–4. doi:10.1001/jama.2013.281053

39 Anderson AP, Mayer MD, Fellows AM, *et al*. Relaxation with immersive natural scenes presented using virtual reality. *Aerosp Med Hum Perform* 2017;88:520–6. https://doi.org/10.3357/amhp.4747.2017

40 Brown P, Waite F, Lambe S, *et al*. Virtual reality cognitive therapy in inpatient psychiatric wards: protocol for a qualitative investigation of staff and patient views across multiple national health service sites. *JMIR Res Protoc* 2020;9:e20300. https://doi.org/10.2196/20300

41 Sanchez-Vives MV, Slater M. From presence to consciousness through virtual reality. *Nat Rev Neurosci* 2005;6:332-339. https://doi.org/10.1038/nrn1651

42 Valmaggia LR, Day F, Rus-Calafell M. Using virtual reality to investigate psychological processes and mechanisms associated with the onset and

For peer review only

maintenance of psychosis: a systematic review. *Soc Psychiatry Psychiatr Epidemiol* 2016;51:921–36. https://doi.org/10.1007/s00127-016-1245-0

43 Browning MH, Mimnaugh KJ, van Riper CJ, *et al*. Can simulated nature support mental health? Comparing short, single-doses of 360-degree nature videos in virtual reality with the outdoors. *Front Psychol* 2020;10:2667. https://doi.org/10.3389/fpsyg.2019.02667

44 Lindner P, Miloff A, Hamilton W, *et al*. Creating state of the art, next-generation Virtual Reality exposure therapies for anxiety disorders using consumer hardware platforms: design considerations and future directions. *Cogn Behav Ther* 2017;46:404–20. https://doi.org/10.1080/16506073.2017.1280843

45 Seabrook E, Kelly R, Foley F, *et al*. Understanding how virtual reality can support mindfulness practice: mixed methods study. *J Med Internet Res* 2020;22:e16106. https://doi.org/10.2196/16106

46 Lindner P, Rozental A, Jurell A, *et al*. Experiences of gamified and automated virtual reality exposure therapy for spider phobia: qualitative study. *JMIR Serious Games* 2020;8:e17807. https://doi.org/10.2196/17807

47 Færden A, Bølgen B, Løvhaug L, *et al*. Patient satisfaction and acute psychiatric inpatient treatment. *Nord J Psychiatry* 2020;74:577–84. https://doi.org/10.1080/08039488.2020.1764620

48 Lindberg MH, Samuelsson M, Perseius KI, *et al*. The experiences of patients in using sensory rooms in psychiatric inpatient care. *Int J Ment Health Nurs* 2019;28:930–9. https://doi.org/10.1111/inm.12593

49 Wykes T, Csipke E, Williams P, *et al*. Improving patient experiences of mental health inpatient care: a randomised controlled trial. *Psychol Med* 2018;48(3):488–97. https://doi.org/10.1017/S003329171700188X

For peer review only

50 Appel L, Kisonas E, Appel E, *et al*. Administering virtual reality therapy to manage behavioral and psychological symptoms in patients with dementia admitted to an acute care hospital: results of a pilot study. *JMIR Form Res* 2021;5:e22406. https://doi.org/10.2196/22406

FIGURE LEGEND

Figure 1 Screenshots from the virtual reality calm room.

For peer review only

For peer review only

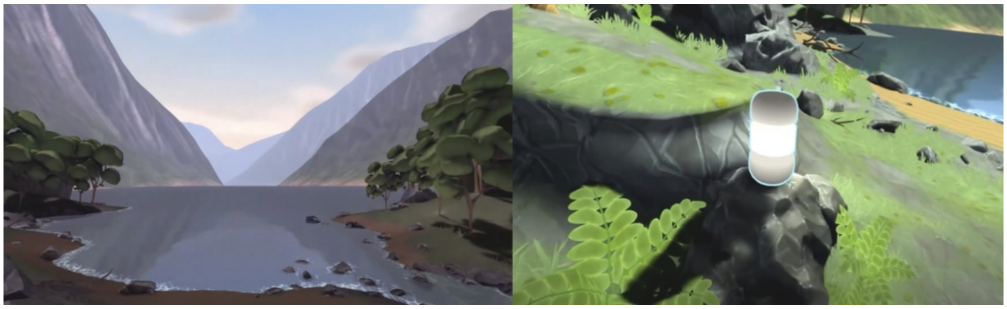

299x90mm (300 x 300 DPI)

COREQ (COnsolidated criteria for REporting Qualitative research) Checklist

Developed from: Tong A, Sainsbury P, Craig J. Consolidated criteria for reporting qualitative research (COREQ): a 32-item checklist for interviews and focus groups. *International Journal for Quality in Health Care*. 2007. Volume 19, Number 6: pp. 349 – 357

Topic	Guide questions/description	Reported on Page
**Domain 1: Research team and reflexivity**		
Personal Characteristics		
1. Interviewer/facilitator	Which author/s conducted the interview or focus group?	8
2. Credentials	What were the researcher's credentials? E.g. PhD, MD	1
3. Occupation	What was their occupation at the time of the study?	1
4. Gender	Was the researcher male or female?	N/A
5. Experience and training	What experience or training did the researcher have?	N/A
Relationship with participants		
6. Relationship established	Was a relationship established prior to study commencement?	N/A
7. Participant knowledge of the interviewer	What did the participants know about the researcher? e.g. personal goals, reasons for doing the research	8
8. Interviewer characteristics	What characteristics were reported about the inter viewer/facilitator? e.g. Bias, assumptions, reasons and interests in the research topic	8
**Domain 2: study design**		
Theoretical framework		
9. Methodological orientation and Theory	What methodological orientation was stated to underpin the study? e.g. grounded theory, discourse analysis, ethnography, phenomenology, content analysis	7
Participant selection		
10. Sampling	How were participants selected? e.g. purposive, convenience, consecutive, snowball	7
11. Method of approach	How were participants approached? e.g. face-to-face, telephone, mail, email	7-8
12. Sample size	How many participants were in the study?	7
13. Non-participation	How many people refused to participate or dropped out? Reasons?	7
Setting		

For peer review only

14. Setting of data collection	Where was the data collected? e.g. home, clinic, workplace	7
15. Presence of non-participants	Was anyone else present besides the participants and researchers?	8
16. Description of sample	What are the important characteristics of the sample? e.g. demographic data, date	10
Data collection		
17. Interview guide	Were questions, prompts, guides provided by the authors? Was it pilot tested?	8
18. Repeat interviews	Were repeat inter views carried out? If yes, how many?	N/A
19. Audio/visual recording	Did the research use audio or visual recording to collect the data?	8
20. Field notes	Were field notes made during and/or after the inter view or focus group?	N/A
21. Duration	What was the duration of the inter views or focus group?	8
22. Data saturation	Was data saturation discussed?	4,9,22
23. Transcripts returned	Were transcripts returned to participants for comment and/or correction?	N/A
**Domain 3: analysis and findings**		
Data analysis		
24. Number of data coders	How many data coders coded the data?	9
25. Description of the coding tree	Did authors provide a description of the coding tree?	9
26. Derivation of themes	Were themes identified in advance or derived from the data?	9
27. Software	What software, if applicable, was used to manage the data?	N/A
28. Participant checking	Did participants provide feedback on the findings?	N/A
Reporting		
29. Quotations presented	Were participant quotations presented to illustrate the themes/findings? Was each quotation identified? e.g. participant number	12-19
30. Data and findings consistent	Was there consistency between the data presented and the findings?	12-19
31. Clarity of major themes	Were major themes clearly presented in the findings?	11
32. Clarity of minor themes	Is there a description of diverse cases or discussion of minor themes?	11

For peer review only
